# Recircumscription and taxonomic revision of *Siderasis*, with comments on the systematics of subtribe Dichorisandrinae (Commelinaceae)

**DOI:** 10.3897/phytokeys.83.13490

**Published:** 2017-07-13

**Authors:** Marco O. O. Pellegrini, Robert B. Faden

**Affiliations:** 1 Universidade de São Paulo, Departamento de Botânica, Rua do Matão 277, CEP 05508-900, São Paulo, SP, Brazil; 2 Jardim Botânico do Rio de Janeiro, Rua Pacheco Leão 915, CEP 22460-030, Rio de Janeiro, RJ, Brazil; 3 Smithsonian Institution, NMNH, Department of Botany, MRC 166, P.O. Box 37012, Washington D.C. 20013-7012, USA

**Keywords:** Atlantic Forest, Brazil, Commelinales, Neotropical flora, spiderwort, Tradescantieae

## Abstract

A new circumscription and a total of six microendemic species, four of them new to science, are herein presented for *Siderasis*, based on field and herbaria studies, and cultivated material. We provide an identification key to the species and a distribution map, description, comments, conservation assessment, and illustration for each species. Also, we present an emended key to the genera of subtribe Dichorisandrinae, and comments on the morphology and systematics of the subtribe.

## Introduction

*Siderasis* Raf. is currently applied to a small genus of neotropical Commelinaceae, comprising only two microendemic species, restricted to the Atlantic Forest of Southeastern Brazil (Pellegrini 2017). It was originally described by Rafinesque (1836), together with several other small genera, in order to better organize the many species misplaced in *Commelina* L. and *Tradescantia* L. Rafinesque (1836) mentioned a possible affinity between *Siderasis*, *Callisia* Loefl. and *Etheosanthes* Raf. (= *Belosynapsis* Hassk.), and considered *Siderasis* not at all similar to *Tradescantia*; but gave no explanation for any of these statements. He also considered *T. fuscata* Lodd. a synonym of his newly described *S. acaulis* Raf., which was characterized by its rusty hirsute indumentum covering the entire plant, short stems, flowers emerging from the roots, petals basally connate, dimorphic stamens varying from four to six, and gynoecium 2–3-locular [sic]. After Rafinesque’s publication, *Siderasis* was completely overlooked by all Commelinaceae specialists for the next 120 years (Moore 1956). In the meantime, Hasskarl (1869) described the new genus *Pyrrheima* Hassk. following his discussions with Schlechtendal during the Botany Meeting in Amsterdam in April of 1865. Hasskarl and Schlechtendal believed that *Pyrrheima* diverged greatly from *Tradescantia* and *Tinantia* Scheidw. due to its non-tubular perianth, six equal and fertile stamens, and lunate anther sacs, and thus merited distinct generic status. Clarke (1881), in his monograph for Commelinaceae, accepted *Pyrrheima*, including only *P. loddigesii* Hassk., and reducing *P. minus* Hassk. to a variety of it. Brückner (1930), noticing that *P. loddigesii* was an unnecessary replacement name for *T. fuscata*, made the new combination *P. fuscata* (Lodd.) G.Brückn., but was unsure if *Siderasis* and *Pyrrheima* were indeed congeneric. This was later confirmed by Moore (1956), when he merged the two by transferring *P. fuscata* to *Siderasis*.

After further period of neglect, the genus was placed in subtribe Dichorisandrinae (Faden and Hunt 1991), along with its sister-genus *Dichorisandra* J.C.Mikan, *Cochliostema* Lem., *Geogenanthus* Ule, and *Plowmanianthus* Faden & C.R.Hardy (Faden and Hunt 1991; Hardy 2001; Evans et al. 2003; Hardy and Faden 2004). However, resolution of the relationships within the group remains elusive. While it appears certain that *Siderasis* is proximally related to *Dichorisandra*, the subtribe as a whole has been recovered as paraphyletic in most molecular and morphological phylogenies to date. Two separate clades are recovered with one containing *Dichorisandra* and *Siderasis* (i.e. subtribe Dichorisandrinae *s.s*), while the remaining three genera (*Geogenanthus* and *Cochliostema*+*Plowmanianthus*) are recovered as one of the early-diverging clades in tribe Tradescantieae (Hardy 2001; Evans et al. 2003; Wade et al. 2006; Zuiderveen et al. 2011; Hertweck and Pires 2014; Pellegrini 2017; Pellegrini et al., in prep.). *Siderasis* has hitherto been characterized by the rusty to bright red hirsute indumentum covering the entire plant (except the petals and androecium), its ebracteolate cincinni, filaments three to four times longer than the anthers, anthers with rimose dehiscence (Hardy and Faden 2004), and exarillate seeds (Faden 1998).

Composition of the genus itself also remains unclear with Faden and Hunt (1991) mentioning two *Siderasis* species, while Faden (1998) mentions two to three species. Barreto (1997), in a survey of the Commelinaceae native to Brazil accepts only *S. fuscata* and reaffirmed *Siderasis* as a monospecific genus. Pellegrini (2017) recently described a new species of *Siderasis*, and provided important information regarding inflorescence and seed morphology in the genus. Clearly, further studies were still necessary to solve the ongoing issues (Pellegrini et al. 2013), and with this in mind recent field and herbaria studies have been undertaken to shed further light on this genus. In an attempt to clarify the taxonomy and systematics of neotropical Commelinaceae, and as part of the authors’ ongoing studies in subtribe Dichorisandrinae (Hardy and Faden 2004; Aona et al. 2012; Aona et al. 2016; Pellegrini and Almeida 2016; Pellegrini 2017), we recircumscribe and revise *Siderasis*, with the description of four new species. We also provide detailed comments on the morphology and systematics of subtribe Dichorisandrinae *s.l.*

## Methods

The description of the species, phenology and illustrations were based on herbaria (A, ALCB, B, BHCB, BHZB, BM, BOTU, BRIT, C, CAL, CEPEC, CESJ, CGE, CNMT, CVRD, ESA, F, FCAB, FLOR, FURB, G, GUA, HAMAB, HAS, HB, HBR, HRB, HRCB, HSTM, HUEFS, HUFSJ, HURB, IAC, ICN, INPA, K, L, MBM, MBML, MG, MO, MY, NY, P, PMSP, PORT, R, RB, RFA, RFFP, SP, SPF, U, UEC, UPCB, US, and WAG; herbaria acronyms according to Thiers, continuously updated), spirit, fresh, and cultivated material. Specimens of *Siderasis albofasciata* M.Pell., *S. almeidae* M.Pell. & Faden sp. nov., and *S. fuscata* were kept in cultivation at the greenhouse of the Jardim Botânico do Rio de Janeiro, in order to observe, photograph, and analyze fresh flowers, fruits, and seeds as well as other phenological data. Fresh specimens, field notes, photographs, and specimens for cultivation were gathered during several field trips across the Brazilian Atlantic Forest, from the states of Sergipe to Rio Grande do Sul, between 2008–2016. Field data and description of *S. medusoides* M.Pell. & Faden sp. nov., and *S. zorzanellii* M.Pell. & Faden sp. nov. were complemented with notes, photographs and spirit samples kindly provided by the collectors. Fertile specimens were deposited in RB, and whenever possible duplicates were sent to US. Indumentum and shape terminology follows Radford et al. (1974); the inflorescence and general morphology terminology follows Weberling (1965, 1989) and Panigo et al. (2011); the fruit terminology follows Spjut (1994); and the seed terminology follows Faden (1991). The conservation assessments followed the recommendations of "IUCN Red List Categories and Criteria, Version 3.1" (IUCN 2001). GeoCAT (Bachman et al. 2011) was used for calculating the Extent of Occurrence (EOO) and the Area of Occurrence (AOO). The distribution of the species is based on herbaria materials, field data, and literature. The classification of vegetation patterns follows IBGE (2012).

## Results

In the present study, we accept six species of *Siderasis*, with four of them newly described here. All species in the genus are microendemics, restricted to the Atlantic Forest of eastern Brazil. Both *Dichorisandra* and *Siderasis* share considerable variation in growth form, inflorescence architecture and androecium arrangement, which may have hindered the emergence of a stable taxonomy. Due to the variation and peculiar morphology of the newly described species, especially the two climbing species, *Siderasis* is recircumscribed below. The genus can be distinguished from the remaining Dichorisandrinae *s.l.* based on floral morphology, especially androecium and microstigmatic morphology. An updated identification key for the genera of Dichorisandrinae *s.l.* is presented, along with comments on the morphology of *Siderasis* compared to the remaining genera of the subtribe.

### Emended key to the genera of Dichorisandrinae *s.l.* (modified from Hardy and Faden 2004)

**Table:** 

1.	Petals with glabrous margins, rarely ciliate with non-moniliform hairs; filaments glabrous, anther sacs not appressed to each other (if appressed, anther sacs not semicircular); capsules thick-walled; seeds arillate	**2**
–	Petals with margins fringed with moniliform hairs; filaments bearded with moniliform hairs, rarely glabrous, anther sacs appressed to each other; capsules thin-walled; seeds exarillate	**3**
2.	Stamens 5–6, staminodes sometimes present; anthers basifixed, anthers sacs parallel, elongate, 3 to 4 times longer than the filaments, connectives inconspicuous, dehiscence poricidal or introrsely rimose, but functionally poricidal; stigmatic papillae multicellular, completely concealing the stylar canal	***Dichorisandra* J.C.Mikan (Fig. 1D–L)**
–	Stamens 6, staminodes absent; anthers dorsifixed, anther sacs divergent, semicircular, 3 to 4 times shorter than the filaments, connectives expanded, dehiscence extrorsely rimose; stigmatic papillae unicellular, restricted to margins of the stigma and leaving the stylar canal evident	***Siderasis* Raf. emend. M.Pell. & Faden (Fig. 1A–C)**
3.	Dracaenoid herbs; roots with terminal tubers; shoots determinate; inflorescences borne at the lower nodes below the leaves; pedicel with glandular hairs, stamens 5–6, all fertile, stigmas never fringed with moniliform hairs	***Geogenanthus* Ule (Fig. 1N)**
–	Rosette herbs; roots without terminal tubers; shoots indeterminate; inflorescences borne among the leaves; pedicels with eglandular hairs, fertile stamens 3, on the upper half of the flower, staminodes 3 (sometimes microscopic), on the lower half of the flower, stigmas commonly marginally fringed with moniliform hairs	**4**
4.	Tank-forming or creeping rosettes, epiphytes, rarely terrestrial; inflorescence a many-branched thyrse, with alternate or verticillate cincinni, cincinni bracts showy; fertile anthers spirally-coiled, hidden within a hood-like structure; testa smooth, sticky when hydrated	***Cochliostema* Lem. (Fig. 1M)**
–	Rosettes not tank-forming, terrestrial; inflorescence reduced to a solitary pedunculate cincinnus, cincinnus bract inconspicuous; fertile anthers semicircular, not hidden within a hood-like structure; testa rugose to foveolate, farinose	***Plowmanianthus* Faden & C.R.Hardy (Fig. 1O)**

#### Siderasis

Taxon classificationPlantaeCommelinalesCommelinaceae

Raf., Fl. Tellur. 3: 67. 1837, emend. M.Pell. & Faden

Pyrrheima Hassk., Flora 52: 366. 1869, nom. illeg. Type species (designated here). P. loddigesii Hassk., nom. illeg. [≡ S. fuscata (Lodd.) H.E.Moore].

##### Type species.

*Siderasis acaulis* Raf. [≡ *S. fuscata* (Lodd.) H.E.Moore].

##### Description.

***Herbs or vines***, perennial, with a definite base, terrestrial or rupicolous. ***Roots*** thin, fibrous, sometimes forming terminal, small, fusiform to oblongoid tubers. ***Rhizomes*** present or not, if present short, shallowly to deeply buried in the ground, rarely only covered by leaf litter. ***Subterraneous stems*** present or not, when present buried deep in the soil, unbranched, produced directly from the short rhizome; internodes moderately elongate to elongate. ***Aerial stems*** with determinate or indeterminate growth, elongated or short to inconspicuous, densely branched or unbranched, when densely branched primary shoot determinate or not, when present secondary shoots determinate; internodes inconspicuous to weakly elongate, or elongate; flagelliform-shoots (ramets) present or not, if present produced after the fertile period, forming a new rosette at the apex, axillary, unbranched, internodes elongate. ***Leaves*** spirally-alternate or distichously-alternate, congested at the apex of the stems forming a rosette or evenly distributed along the secondary branches, sessile to subpetiolate or petiolate, sheathing at the base, ptyxis involute; blades membranous to chartaceous or succulent, base symmetric or slightly to completely asymmetric, margins slightly revolute to flat, apex curved or straight. ***Synflorescence*** composed of a solitary main florescence or with 1–7 coflorescences. ***Main florescence (inflorescence)*** a thyrse, terminal or apparently so, rarely axillary, a many-branched, pedunculate thyrse, with alternate cincinni or reduced to a solitary pedunculate cincinnus; basal bract sessile or amplexicaulous or sheathing; cincinni bracts sessile or amplexicaulous; cincinni pedunculate, 1–many-flowered; bracteoles present or not. ***Flowers*** bisexual or staminate, actinomorphic or zygomorphic, chasmogamous, flat, pedicellate or sessile; pedicels erect during pre-anthesis and anthesis, erect or deflexed post-anthesis, generally elongating in fruit; sepals 3, unequal, free, membranous or fleshy, persistent and accrescent in fruit, the uppermost external, broader than the others, sometimes also shorter than the others; petals 3, deliquescent, free, margins entire to irregularly lacerated, glabrous, rarely ciliated with non-moniliform hairs, apex entire to irregularly lacerated, subequal, the lowermost either broader or longer than the others; stamens 6, equal or unequal, straight or curved upwards, filaments free, glabrous, straight or sigmoid, anthers dorsifixed, extrorsely rimose, anther sacs semicircular, divergent, pollen white, connectives expanded, quadrangular to rectangular; ovary sessile, globose to broadly oblongoid to ellipsoid in outline, trigonous with obtuse to round angles in cross-section, densely hirsute or lanate or velutine, 3-locular, locules equal, 3–6-ovulate, ovules hemianatropous, biseriate to partially uniseriate; style terminal, straight or curved upwards; stigma annular-truncate or annular-capitate, marginally papillate leaving the stylar canal evident, papillae unicellular. ***Capsules*** loculicidal, thick-walled, 3-valved, globose or subglobose to broadly ellipsoid to broadly oblongoid to oblongoid in outline, trigonous with obtuse to round angles in cross-section, smooth to sparsely reticulate, apiculate due to persistent style base. ***Seeds*** (1–)3–6 per locule, arillate, obconic to ellipsoid, dorsiventrally compressed, ventrally slightly flattened or with a mild ridge, testa foveolate or rugose; hilum C-shaped, in a shallow depression; embryotega semidorsal or semilateral, relatively inconspicuous, without a prominent apicule; aril cream-colored to hyaline, slightly to completely translucent, thick or inconspicuous.

##### Etymology.

*Siderasis* was named in allusion to the peculiar red to bright-red hairs that cover almost the entire plant, but especially the leaves. However, only *S. fuscata* possesses the aforementioned hairs, and all of the remaining species possess leaf blades covered by hyaline to light brown, rarely rusty hairs.

##### Habitat, distribution and ecology.

*Siderasis* is endemic to the Atlantic Forest domain in coastal Brazil, occurring in the states of Bahia, Espírito Santo, and Rio de Janeiro (Fig. 2). More specifically, *Siderasis* is restricted to the Central Corridor of the Atlantic Forest, growing in remnants of semideciduous forests associated with inselbergs, between 90–1350 m above sea level. The genus is composed exclusively by microendemic species distributed in very small and fragmented subpopulations, susceptible to deforestation and illegal collection of specimens for ornamental purposes.

##### Biogeography.

Since most phylogenies for Commelinaceae corroborate the paraphyly of Dichorisandrinae (Evans et al. 2000, 2003; Hardy 2001; Wade et al. 2006; Zuiderveen et al. 2011; Hertweck and Pires 2014; Pellegrini et al., in prep.), we can hypothesize on the independent diversification of these lineages from a biogeographical point of view. The clade composed by *Cochliostema*, *Geogenanthus*, and *Plowmanianthus* is consistently recovered as the second lineage to diverge in tribe Tradescantieae, following the diversion of subtribe Streptoliriinae (Evans et al. 2003; Wade et al. 2006; Zuiderveen et al. 2011; Hertweck and Pires 2014; Pellegrini et al., in prep.). The ancestor of this lineage probably originated in the Amazon Basin, and posteriorly diversified in the Guyana Shield, northern Andes, and Central America reaching Costa Rica (Hardy 2001; Hardy and Faden 2004). On the other hand, the clade composed by *Dichorisandra* and *Siderasis* is recovered as the third lineage to diverge in Tradescantieae (Evans et al. 2003; Wade et al. 2006; Zuiderveen et al. 2011; Hertweck and Pires 2014; Pellegrini et al., in prep.). The ancestor of this clade probably originated and diversified in the Atlantic Forest domain, since it is the center of diversity of both genera. Subsequently, the ancestors of various *Dichorisandra* lineages might have dispersed, more than once, diversifying in the Amazon Basin through gallery forests in the Cerrado domain.

##### Growth form and leaf morphology.

*Siderasis* possesses two clearly differentiated growth patterns: (1) rosette herbs, generally with very short internodes, and spirally-alternate, symmetrical leaves (Fig. 3A–B); (2) climbing vines, with elongated internodes, and distichously-alternate, asymmetrical leaves (Fig. 3C–D). The rosette habit has hitherto been the only one recognized in the genus. Faden (1998) mentioned the existence of a climbing species in the genus, but due to the synoptic nature of that publication, no further remarks were made on the subject. The climbing habit is relatively uncommon in the family, but found in the closely related *Dichorisandra*. However, in *Dichorisandra* the plants tend to lean on nearby trees and shrubs, later producing pendant branches, or even growing completely intertwined with more robust shrubs (Fig. 3E–F). In *Siderasis*, the primary branch grows at the base of a tree (Fig. 3C), posteriorly spirally ascending around the trunk, and finally producing the flowering secondary branches (Fig. 3D). In the remaining genera of Dichorisandrinae, growth form is stable, with almost no variation within each genus. In *Cochliostema*, the plants tend to be tank-forming rosette herbs, but creeping individuals are also known in *C. velutinum* Read (Hardy 2001). In *Geogenanthus*, the plants always possess a dracaenoid habit, with leaves congested at the apex (Hardy 2001). In *Plowmanianthus*, the plants are always rosette herbs with very short stems (Hardy and Faden 2004).

Considerable variation in leaf morphology occurs in *Siderasis*, with leaves ranging from: (1) sessile to subpetiolate (Fig. 3B–D); (2) truly petiolate, as in *S. fuscata* (Fig. 3A, 8C). Truly petiolate leaves are extremely rare in Commelinaceae, being recorded only in a handful of species restricted to the peculiar-looking subtribe Streptoliriinae, mostly comprised of vining plants (Pellegrini and Faden, pers. observ.). Phyllotaxy in *Siderasis* can range from distichous to spirally-alternate, the arrangement being correlated to symmetry of the leaf blades.

##### Inflorescence morphology.

In all *Dichorisandra* and two species of *Siderasis* (i.e. *S. spectabilis* and *S. zorzanellii*), the main florescence is a many-branched, pedunculate, terminal or axillary thyrse with alternate cincinni, each cincinnus being multi-flowered. In the remaining species of *Siderasis* (i.e. *S. albofasciata*, *S. almeidae*, *S. fuscata* and *S. medusoides*), the main florescence is composed of a thyrse reduced to a solitary cincinnus, as described in Pellegrini (2017; Fig. 4A). These reduced thyrsi are arranged into a synflorescence that may contain up to seven coflorescences. The center of the mature *Siderasis* rosette may contain several terminal or apparently terminal synflorescences. In *Dichorisandra* and the two climbing species of *Siderasis*, the main axis of the inflorescence is usually well developed, thus producing a typical looking thyrse (Fig. 4B). Nevertheless, the inflorescences may also be extremely reduced in some species (i.e. *D. acaulis* group), due to the shortening of the inflorescence’s internodes (Pellegrini and Almeida 2016). The cincinni are also very short (i.e. sessile to subsessile), being enclosed by the leaf sheaths and not obvious at first glance (Pellegrini and Almeida 2016). The flowers are peculiarly long-pedicellate, giving the impression that all flowers emerge directly from the apex of the stems (Pellegrini and Almeida 2016; Fig. 4C). Despite the extreme reduction and superficial similarity, this inflorescence pattern differs from the one found in the rosette species of *Siderasis*, since it still is a many-branched thyrse. In *Plowmanianthus* the main florescence is also reduced to a solitary cincinnus. Nonetheless, coflorescences only develop after the main florescence has failed to develop or set fruit, and the cincinni from the primary and secondary thyrsi are morphologically distinct (Hardy and Faden 2004). In *Geogenanthus* the inflorescences are always born at the base of the plant, near the ground. Aside from that, the main florescence is a pedunculate, fascicle-like thyrse, with (1–)2–4–several alternate cincinni (Hardy 2001). Finally, in *Cochliostema* the main florescence is a many-branched, pedunculate, axillary thyrse, with alternate to verticillate cincinni, each cincinnus being multi-flowered and subtended by showy and cucullate spathaceous bracts (Hardy and Stevenson 2000; Hardy 2001).

##### Floral symmetry.

**Figure 1.**
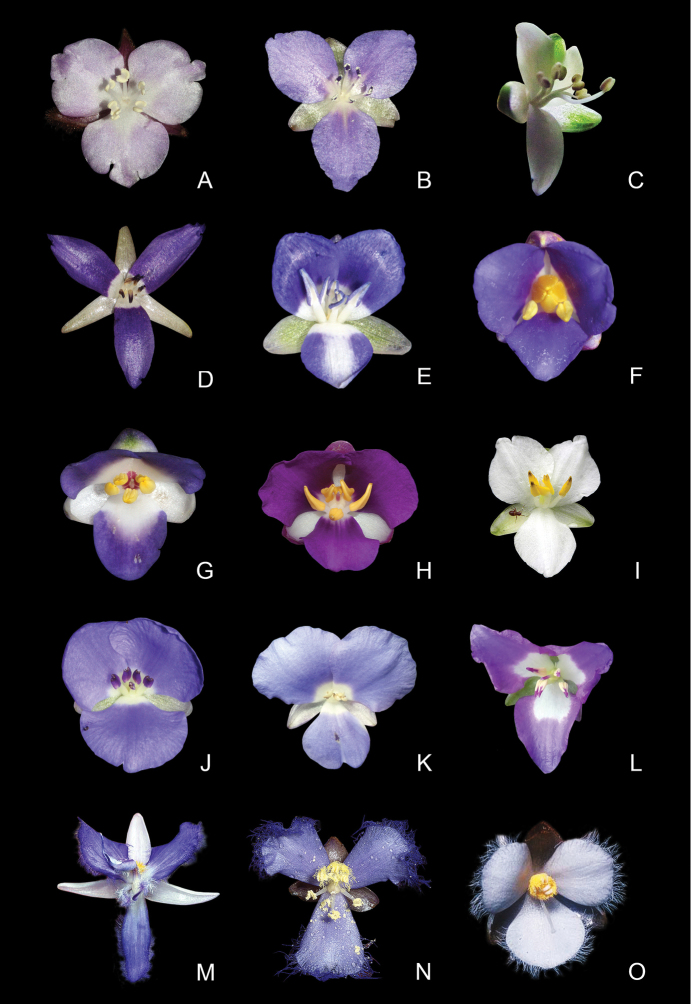
Floral morphology of subtribe Dichorisandrinae *s.l.* **A–C** *Siderasis* Raf. emend M.Pell. & Faden: **A** *S. fuscata* (Lodd.) H.E.Moore **B** *S. albofasciata* M.Pell. **C** *S. zorzanellii* M.Pell. & Faden. **D–L** *Dichorisandra* J.C.Mikan: **D** *D. acaulis* Cogn. **E** *D. hexandra* (Aubl.) C.B.Clarke **F** *D. thyrsiflora* J.C.Mikan **G** *D. paranaënsis* D.Maia et al. **H** *D. nana* Aona & M.C.E.Amaral **I** *D. incurva* Mart. **J** *D. penduliflora* Kunth **K** *D. sagittata* Aona & M.C.E.Amaral **L** *D. radicalis* Nees & Mart. **M** *Cochliostema odoratissimum* Lem. **N** *Geogenanthus rhizanthus* (Ule) G.Brückn. **O** *Plowmanianthus panamensis* Faden & C.R.Hardy. Photographs **A–B, D–G, J** by M.O.O. Pellegrini, **C** by J.P.F. Zorzanelli, **H** by V. Bittrich, **I** by G.H. Shimizu, **K** by J.L. Costa-Lima, **L** by M.A.N. Coelho, **M** by R. Moran, **N** by D. Scherberich, and **O** by C.R. Hardy.

**Figure 2.**
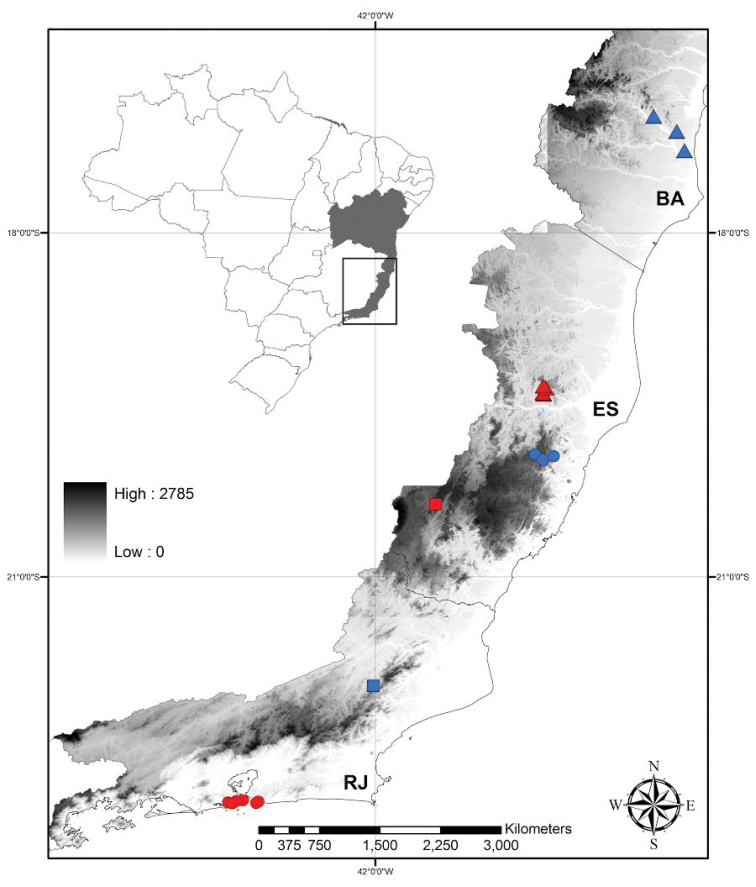
Distribution map of *Siderasis* Raf. *emend.* M.Pell. & Faden. **Blue circles**– *S. albofasciata*; **Blue triangles**– *S. almeidae*; **Red circles**– *S. fuscata*; **Red triangles**– *S. medusoides*; **Blue square**– *S. spectabilis*; **Red Square**– *S. zorzanellii*; **BA**– state of Bahia; **ES**– estate of Espírito Santo; **RJ**– estate of Rio de Janeiro.

**Figure 3.**
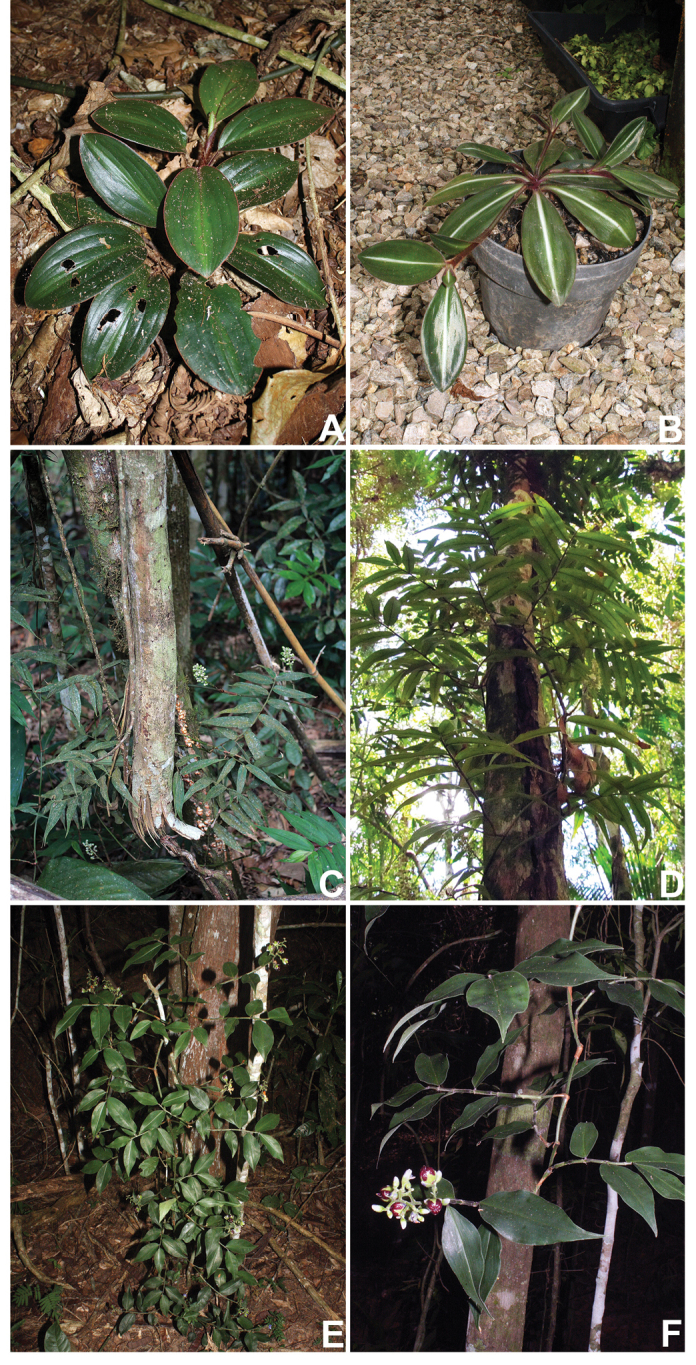
Growth forms in Dichorisandrinae *s.s.* **A–D** *Siderasis* Raf. emend. M.Pell. & Faden: **A** rosette habit of *S. fuscata* (Lodd.) H.E.Moore **B** rosette habit with flagelliform-shoots of *S. albofasciata* M.Pell. **C** habit of an immature, still prostrate, individual of *S. zorzanellii* M.Pell. & Faden. **D** habit of a mature individual of *S. zorzanellii* spirally ascending a tree. **E–F** *Dichorisandra hexandra* (Aubl.) C.B.Clarke: **E** intertwined habit **F** decumbent stem apex, bearing flowers and fruits. Photographs **A–B, E–F** by M.O.O. Pellegrini, **C–D** by J.P.F. Zorzanelli.

Two distinct floral patterns can be observed in different species of *Siderasis*: (1) flowers are always bisexual, actinomorphic, having 6 equal stamens arranged cyclically around the ovary, with straight filaments (Fig. 1A–B); (2) flowers bisexual or staminate, zygomorphic, having 6 unequal stamens curved upwards, with sigmoid filaments (Fig. 1C). Furthermore, in the zygomorphic staminate flowers, the lower antepetalous stamen is longer, and is arranged and curved in the same way as the style in bisexual flowers. The first flower morph is very similar to that found in the *D. acaulis* group (Pellegrini and Almeida 2016; Fig. 1D), while the second is equivalent to that of the *D. hexandra* and *D. incurva* groups (Fig. 1E, I). In *Dichorisandra*, flower symmetry is generally influenced by the positioning of the stamens, rather than by the relationship of stamens and staminodes. Actinomorphic flowers can be found not only in the *D. acaulis* group (Fig. 1D), but also in a group of still-undescribed species from the Guyana Shield (Faden and Pellegrini, pers. observ.). In all remaining species groups in *Dichorisandra*, the flowers are clearly zygomorphic, either due to the number of stamens, their size and/or position. In the *D. thyrsiflora* group, the androecium is generally composed of six fertile stamens, four of them curved towards the center of the flower, and the two lower lateral ones curved towards their opposing sides (Fig. 1F). An exception can be noticed in *D. paranaënsis* D.Maia et al. (Fig. 1G) and *D. nana* Aona & M.C.E.Amaral (Fig. 1H). In *D. paranaënsis* the stamens are curved upwards, varying from five fertile stamens with a staminode (present or not) to six fertile stamens, and introrsely rimose anthers. On the other hand, in *D. nana* the six fertile stamens are curved upwards, and possess poricidal anthers. In the *D. incurva* (Fig. 1I), *D. penduliflora* (Fig. 1J), *D. leucophthalmos* (Fig. 1K), and *D. radicalis* groups (Fig. 1L), the androecium is composed of five stamens (generally with an upper staminode; notice the filiform staminode in Fig. 1L), rarely six fertile stamens, curved upwards, and with introrsely rimose anthers. In the *D. incurva* and *D. leucophthalmos* groups, the anthers are always yellow (Figs 1I, K), while in the *D. penduliflora* and *D. radicalis* groups, the anthers are white, generally with the anther sacs partially to totally colored in blue, pink or purple (Figs 1J, L). The remaining genera of Dichorisandrinae possess strongly zygomorphic flowers, especially due to the position and/or number of stamens: (1) 5–6 dimorphic, free and fertile stamens in *Geogenanthus* (Hardy 2001; Fig. 1N); (2) 3 stamens in the upper side of the flower, fused in a hood-like structure, and 3 lower staminodes (the middle one microscopic) in *Cochliostema* (Hardy 2001; Fig. 1M); (3) and 3 free to partially fused stamens in the upper side of the flower, and 3 lower staminodes (generally all of them microscopic) in *Plowmanianthus* (Hardy and Faden 2004; Fig. 1O).

##### Androecium and gynoecium morphology.

The anthers in *Siderasis* are dorsifixed, with extrorsely rimose dehiscence, two times wider than long, three to four times shorter than the filaments, with semicircular, divergent anthers sacs, and expanded connectives (Fig. 1A–C). In *Dichorisandra* the anthers are basifixed, with poricidal or introrsely rimose (but functionally poricidal) dehiscence, three to four times longer than wide, and three to four times longer than the filaments, rarely equal to the filaments, with elongate, parallel anther sacs, and inconspicuous connectives (Aona 2008; Figs 1D–L). In *Cochliostema*, *Geogenanthus* and *Plowmanianthus* the anthers vary from dorsifixed to basifixed, with extrorsely rimose dehiscence, as wide as long to two times shorter than the filaments, with semicircular to spirally-coiled, appressed anther sacs, and inconspicuous connectives (Hardy and Stevenson 2000; Hardy 2001; Hardy and Faden 2004; Figs 1M–O).

The gynoecium is fairly homogeneous in Dichorisandrinae *s.l.*, with all genera having sessile, 3-locular ovaries, with all locules fertile, ovules hemianatropous, biseriate to partially uniseriate, style terminal, straight or bent at the apex, stigma annular-truncate to annular-capitate, peripherally ciliate with moniliform hairs (i.e. *Cochliostema* and *Plowmanianthus*) or not (i.e. *Dichorisandra*, *Geogenanthus* and *Siderasis*). In *Siderasis*, the stigmatic papillae are unicellular, and restricted to the margins of the stigma, leaving the stylar canal evident (Owens and Kimmins 1981). On the other hand, in *Dichorisandra*, the stigmatic papillae are multicellular, and evenly distributed on the stigma, completely concealing the stylar canal (Owens and Kimmins 1981).

##### Fruit and seed morphology.

The capsules of *Dichorisandra* and *Siderasis* can be differentiated from capsules of other Commelinaceae by their thick and tough walls. In Commelinaceae the fruits are commonly (1–)2–3-locular, thin walled, septicidal capsules (Faden 1998). *Dichorisandra* and *Siderasis* possess 3-locular, 3-valvar capsules, and arillate seeds. The aril in *Dichorisandra* is generally opaque (rarely hyaline), usually thick (rarely inconspicuous), and colored from white to grayish or bright orange (rarely colorless) (Fig. 5A–B). Whereas the aril in *Siderasis* can be hyaline to slightly hyaline, inconspicuous or thick, and cream-colored to colorless (Fig. 5C–D). The seeds in both genera are very similar in gross morphology, varying in shape from obconic to ellipsoid to quadrangular; in ornamentation from foveolate to scrobiculate to rugose, with a semilateral to semidorsal embryotega, and with a C-shaped hilum. In *Cochliostema*, *Geogenanthus* and *Plowmanianthus* the capsules are thin-walled, 3-locular, 3-valvar, and with exarillate seeds. In *Cochliostema* the capsules are narrowly cylindrical, and the seeds vary from subcylindrical to narrowly oblongoid, with a smooth testa that becomes sticky when hydrated, semidorsal embryotega, and a linear hilum with curved edges. In *Geogenanthus* and *Plowmanianthus* the capsules are fusiform to ellipsoid, the seeds range from reniform to ellipsoid, with rugose to foveolate, farinose testa, lateral embryotega, and a C-shaped hilum (Hardy 2001; Hardy and Faden 2004).

In *Dichorisandra* and *Siderasis* capsule and seed morphology differences may have great taxonomic potential. In *Dichorisandra*, many of the aforementioned species groups display characteristic capsule and seed morphology, as exemplified in the *D. acaulis* group by Pellegrini and Almeida (2016). In the *D. thyrsiflora* group, capsule morphology can easily differentiate most known species, based on shape, coloration, texture and pubescence (Pellegrini, pers. observ.). In *Siderasis*, capsule morphology shows a similar potential, with the fruits of *S. zorzanellii* being completely deviant in shape, texture and pubescence from the remaining species. Unfortunately, since the fruits of *S. spectabilis* are still unknown, it is impossible to know if this change in capsule morphology is correlated to the change in habit from rosette to vining herbs. *Siderasis fuscata* possesses unique seed morphology, being the only known species with an inconspicuous and hyaline aril, testa light gray to gray, and foveolate. Field expeditions focused on collecting fruit and seed samples of all species of *Siderasis* could be of great taxonomic value. It is possible that most, if not all, presently accepted species could be differentiated based exclusively on fruit and seed morphology.

##### Reproductive biology.

Little is known regarding the floral biology of subtribe Dichorisandrinae, although this subtribe possesses the greatest range in inflorescence architecture and floral patterns in the family. In *Siderasis* the anthers are always extrorsely rimose, but apart from the floral specialization (i.e. zygomorphic, bisexual or staminate flowers, and unequal and sigmoid stamens) in the two climbing species and the petals with margins ciliated with non-moniliform hairs, a character unique in the family, in *S. spectabilis*, the flowers are relatively unspecialized. *Dichorisandra* possesses a wide variation in flower morphology and androecium arrangement. Its flowers can range from actinomorphic to zygomorphic, the stamens can vary from (5–)6, sometimes with the upper stamen reduced to a staminode in some species. The filaments can be either straight, slightly sigmoid or slightly twisted depending on their position in the flower, while the anthers can be introrsely rimose and functionally poricidal or truly poricidal (Aona 2008; Fig. 1D–L). On the other hand, in *Cochliostema*, *Geogenanthus* and *Plowmanianthus*, the flowers are highly specialized, being zygomorphic (in all genera), scented (in *Cochliostema*), with a high frequency of cleistogamous flowers (in *Plowmanianthus*), petals and stigma fringed with moniliform hairs (fringed petals in all genera, stigma fringed exclusively in *Cochliostema* and *Plowmanianthus*), filaments bearded with moniliform hairs (in all genera), functionally poricidal androecium (in *Cochliostema*, due to the hood-like structure enclosing the anthers), and curved to spirally-coiled anthers (in all genera) (Hardy and Stevenson 2000; Hardy 2001; Hardy and Faden 2004; Fig. 1M–O). Only three species of *Dichorisandra* have had their reproductive biology investigated, presenting typical buzz-pollination, performed by bumblebees (Apidae) and/or sweatbees (Halictidae) (*D. thyrsiflora*, Boaventura and Matthes 1987; *D. hexandra* and *D. incurva*, Sigrist and Sazima 2015). Information regarding flower visitation in *Cochliostema*, *Geogenanthus*, and *Plowmanianthus* is completely lacking from the available literature. During our field studies and while observing the *Siderasis* specimens grown at the greenhouse of Jardim Botânico do Rio de Janeiro, the first author has observed flowers of *S. albofasciata*, *S. almeidae*, and *S. fuscata* being visited by stingless honey bees (Apidae, tribe Meliponini). *Siderasis medusoides* was not seen in the field, but high-resolution photographs sent by one of the collectors clearly show several small ants walking around the flowers and cincinni (Fig. 9C). The bees might either represent pollen robbers or potential pollinators, but the presence of the ants is hard to explain, since nectaries are unknown for Commelinaceae (Faden 1992, 1998). Further studies on the reproductive biology of *Siderasis* are clearly needed.

Aside from the peculiar floral diversity, Dichorisandrinae *s.l.* has two genera (out of five) and the majority of species in the family with arillate seeds (Pellegrini 2017). Nonetheless, no study has ever focused on vector-mediated (i.e. zoo-choric) seed dispersal in the family. In *Dichorisandra*, the seeds in the *D. hexandra* group are most certainly dispersed by birds (Faden 1992), due to the plants vining habit (Fig. 3E), which help in displaying the seeds, covered by an orange to bright orange, thick and opaque aril (Fig. 5B). The seeds in the *D. thyrsiflora* group are covered by a thick and opaque, white to cream-colored aril (Fig. 5A), and are generally easy to see in the field, due to the plants high stature (Pellegrini pers. observ.). Nonetheless, these species lack the characteristic colors that are generally associated with bird pollination/dispersal (i.e. pink, red, orange and yellow; Fleming and Estrada 1993), always present in the *D. hexandra* group. The species in the *D. acaulis* group possess seeds also covered by a thick and opaque, white aril, lacking the visual attraction associated with bird dispersal, and also lack an elevated display, since they are always shorter than 1 m long (Pellegrini and Almeida 2016). These seeds might be dispersed by ants, or by small terrestrial vertebrates (e.g. small rodents), instead of being dispersed by birds, as hypothesized for other species of *Dichorisandra*. The seeds from the rosette species of *Siderasis* have similar morphological and ecological features to the species from the *D. acaulis* group. These species also have small stature and seeds with hyaline and inconspicuous, or cream-colored, slightly translucent, thick arils (Fig. 5C–D), being most probably dispersed by animals similar to the ones dispersing the seeds of the species in the *D. acaulis* group.

From a phylogenetic point of view, it seems that vector-mediated seed dispersal has evolved several times in the family: (1) arillate seeds are recorded for *Dichorisandra* and *Siderasis* in Dichorisandrinae, *Amischotolype* Hassk., *Coleotrype* C.B.Clarke and *Porandra* Hong in Coleotrypinae (Pellegrini 2017), and *Spatholirion* Ridl. in Streptoliriinae (Thitimetharoch 2004); (2) appendaged seeds are recorded for at least two separate lineages in tribe Commelineae (i.e. some species of *Commelina* L. and *Murdannia* Royle; Pellegrini et al. 2016); (3) truly fleshy fruits are known only from *Palisota* Rchb. *ex* Endl. (Faden 1998); (4) in *Tradescantia zanonia* (L.) Sw. the fleshy sepals cover the indehiscent fruit at post-anthesis, producing a sweet and atro-vinaceous berry-like fruit, dispersed by birds (Pellegrini, obs. pers.); (5) in *Pollia* Thunb. the fruits are dry, crustaceous and indehiscent, and due to their vibrant colors (metallic blue to shiny black) mimic real berries (Faden 1978); (6) in some *Commelina* (i.e. the species originally placed under *Phaeosphaerion* Hassk. and *Commelinopsis* Pichon) the fruits are morphologically similar to those of *Pollia*, being also crustaceous, but either dehiscent or indehiscent (Faden and Hunt 1987); (7) and sticky capsules covered by a mixture of hook and minute glandular hairs, in *Rhopalephora* Hassk. (Pellegrini et al., in prep.). Nonetheless, further investigations are needed to better understand the ecology and evolution of vector-mediated seed dispersal in Commelinaceae.

**Figure 4.**
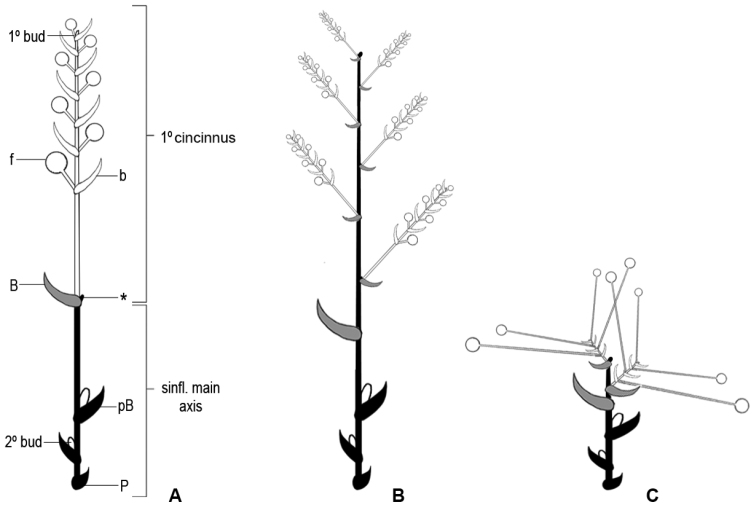
Inflorescence architecture in Dichorisandrinae *s.s.* **A** diagram of the basic *Siderasis* Raf. emend M.Pell. & Faden inflorescence type, consisting of a thyrse reduced to a solitary cincinnus **B** diagram of the basic *Dichorisandra* inflorescence type (also characteristic of *S. spectabilis* and *S. zorzanellii*), consisting of a many-branched thyrse with alternate, many-flowered cincinni **C** diagram of the basic *D. acaulis* species group inflorescence type, where the main florescence axis and cincinni axis are greatly reduced, and the pedicels are peculiarly elongated. **P** = prophyll; **pB** = peduncle bract on main synflorescence axis; * = aborted or dormant apex of main inflorescence axis (usually not observed); **B** = cincinnus bract; **b** = bracteole; **f** = flower; **1°bud** = bud terminating cincinnus; **2°bud** = bud in axil of peduncle bract with potential to develop into a secondary thyrse (coflorescence); Modified from Pellegrini (2017).

**Figure 5.**
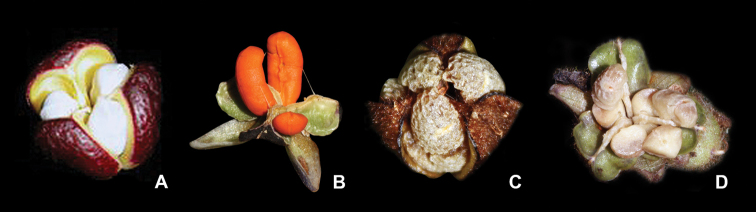
Aril morphology in Dichorisandrinae *s.s.* **A–B** *Dichorisandra* J.C.Mikan: **A** *D. thyrsiflora* J.C.Mikan **B** *D. hexandra* (Aubl.) C.B.Clarke. **C–D** *Siderasis* Raf. emend. M.Pell. & Faden: **C** *S. fuscata* (Lodd.) H.E.Moore **D** *S. albofasciata* M.Pell. Photographs by M.O.O. Pellegrini.

### Key to the species of *Siderasis*

**Table:** 

1.	Vining herbs; aerial stems spirally-twining, densely branched; leaves distichously-alternate, blades ±asymmetric at base; main florescence a many-branched thyrse, with alternate cincinni; flowers bisexual or staminate, zygomorphic, stamens unequal, curved upwards, filaments sigmoid, stigma annular-capitate; embryotega semidorsal	**2**
–	Rosette herbs; aerial stems inconspicuous or trailing, unbranched; leaves spirally-alternate, blades symmetric at base; main florescence reduced to a solitary cincinnus; flowers always bisexual, actinomorphic, stamens equal, directed towards the center of the flower, filaments straight, stigma annular-truncate; embryotega semilateral	**3**
2.	Inflorescences always terminal in the secondary branches; flowers 1–1.3 cm diameter, petals dark mauve to vinaceous, rarely light pink or white, margins ciliate with non-moniliform hairs; northern montane Rio de Janeiro state	***Siderasis spectabilis* M.Pell. & Faden (Fig. 10–11)**
–	Inflorescences axillary in older primary branches and/or terminal in the secondary branches; flowers 0.7–0.9 cm diameter, petals white, margins glabrous; southern montane Espírito Santo state	***Siderasis zorzanellii* M.Pell. & Faden (Fig. 1C, 3B–C & 12)**
3.	Leaves petiolate, indumentum bright red to red, hirsute on both sides; bracteoles absent; capsules ellipsoid to fusiform, greenish brown with 3 atro-vinaceous stripes; seeds grey to light grey, testa foveolate, aril hyaline and inconspicuous; coastal Rio de Janeiro state	***Siderasis fuscata* (Lodd.) M.E.Moore (Fig. 1A & 8)**
–	Leaves sessile to subpetiolate, indumentum rusty to light brown to hyaline, adaxially hispid, abaxially hispid to lanate; bracteoles present; capsules oblongoid to broadly oblongoid to subglobose, green; seeds medium to dark brown, testa rugose, aril cream-colored, slightly translucent and thick; Bahia and Espírito Santo states	**4**
4.	Rosettes forming flagelliform-shoots; leaves adaxially dark green with a thin white to silvery line along the midvein; flowers pedicellate, petals with white proximal third, anthers purple to bluish purple; capsules with elongated pedicels up to 7.2 mm long; central montane Espírito Santo state	***Siderasis albofasciata* M.Pell. (Fig. 1B & 6)**
–	Rosettes not forming flagelliform-shoots; leaves adaxially uniformly green to dark green; flowers sessile, petals evenly colored, anthers white; capsules with elongated pedicels shorter than 2.2 mm long	**5**
5.	Aerial stems with elongate internodes; leaves covered with light brown to rusty hairs, midvein shallowly canaliculate; cincinni compact, straight; sepals fleshy, internally lilac to purple, petals rhomboid to broadly obtrullate, ovary densely lanate; southern Bahia state	***Siderasis almeidae* M.Pell. & Faden (Fig. 7)**
–	Aerial stems with inconspicuous internodes; leaves covered with hyaline hairs, midvein deeply canaliculate; cincinni elongated, tangled; sepals membranous, internally light green, petals obovate to spatulate, ovary hispid; northern lowland Espírito Santo state	***Siderasis medusoides* M.Pell. & Faden (Fig. 9)**

#### 1. Siderasis albofasciata

Taxon classificationPlantaeCommelinalesCommelinaceae

M.Pell., Nordic J. Bot. 35(1): 30. 2017.

Figs 1B , 2 , 6

##### Type.

BRAZIL. Espírito Santo: Santa Teresa, Alto do Julião, Fazenda Novo Triunfo, property of Mrs. Florinda, gallery forest with rocky formations, above the dam, fl., fr., 18 Apr 2013, M.O.O. Pellegrini et al. 337 (holotype: RB barcode RB00813532!; isotype: US!).

##### Description.

***Herbs*** ca. 10 cm tall, rhizomatous, terrestrial or rupicolous. ***Roots*** with terminal tubers present. ***Rhizomes*** buried deep in the ground. ***Subterraneous stems*** with internodes moderately elongate, vinaceous, sparsely lanate, hairs light brown to hyaline. ***Aerial stems*** short to inconspicuous, unbranched; internodes inconspicuous to weakly elongate, vinaceous, lanate, hairs light brown to hyaline; flagelliform-shoots (ramets) present. ***Leaves*** spirally-alternate, forming a rosette at the apex of the aerial stems, sessile to subpetiolate; sheaths 0.7–1.3 cm long, vinaceous, with or without green spots, lanate, hairs light brown to rusty; subpetiole 1–2.7 cm long to inconspicuous, D-shaped in cross section, canaliculate, dark green to vinaceous, hispid, hairs light brown to hyaline; blade (4.5–4.8–)10–15.8 × (3.1–3.5–)4.4–7.2 cm, elliptic to obovate, rarely lanceolate, succulent, adaxially dark green, with a thin white stripe along the midvein, hispid, hairs light brown to hyaline, abaxially vinaceous to atro-vinaceous, lanate, hairs light brown, base slightly subcordate to cuneate, vinaceous, margins vinaceous, slightly revolute, apex acute, straight to curved downwards; midvein adaxially inconspicuous, slightly impressed, abaxially prominent, obtuse, secondary veins 3–5, inconspicuous in both faces, becoming more evident when dry. ***Synflorescence*** composed of a solitary main florescence with 1–2 coflorescences. ***Main florescence (inflorescence)*** reduced to a solitary pedunculate cincinnus, terminal or apparently so; basal bract 11.3–13.4 × 4.8–7.8 mm, triangular, slightly cymbiform, amplexicaulous, vinaceous, hispid, hairs rusty, opaque at the base and margins; inflorescence main axis 2.1–4.4 cm long, vinaceous, densely hispid, hairs rusty to brown; cincinni bract 3.3–6 × 2.2–4.6 mm, triangular, amplexicaulous, vinaceous, hispid, hairs rusty; cincinni (3–)5–8-flowered, peduncles 0.8–1.6 cm long, vinaceous, densely hispid, hairs rusty to brown, reflexed in fruit; bracteoles 2.9–4.4 × 2.8–3.2 mm, broadly ovate to depressed ovate, sessile, revolute, vinaceous to pinkish purple, sparsely hispid, hairs rusty, apex rounded to truncate. ***Flowers*** bisexual, actinomorphic, 2.3–2.8 cm diameter, pedicellate; pedicel 1–7.2 mm long, white to light green, hispid, hairs rusty, reflexed and slightly elongate in fruit; floral buds 0.7–1.6 × 0.3–0.6 cm, ellipsoid to narrowly obovoid, light green, apex obtuse; sepals 0.9–1.1 × 0.4–0.7 cm, narrowly ovate to elliptic, membranous, white to light green on both sides, externally sparsely hispid, hairs hyaline to rusty, rusty in fruit, internally glabrous, margin hyaline, apex obtuse, slightly purple; petals 1.3–1.6 × 1–1.2 cm, broadly ovate to broadly elliptic, bluish lilac to bluish purple, proximal third white, base cuneate, margin entire, apex obtuse to rounded, sometimes irregularly lacerated; stamens equal, filaments 5–7.1 mm long, straight, white, terminal third purple to bluish purple, anthers 1.5–2.2 × 1.3–2 mm, anther sacs purple to bluish purple, connectives quadrangular, purple; ovary 1.5–2 × 1.5–2 mm, globose, white, densely hispid, hairs hyaline, style 4.1–6.3 mm long, straight, white, terminal third purple to bluish purple; stigma annular-truncate, purple to bluish purple, papillate. ***Capsules*** 1–1.3 × 0.7–0.9 cm, subglobose to broadly oblongoid in outline, smooth, green, when mature light brown, hispid, hairs rusty. ***Seeds*** 3.3–5.2 × 2.4–2.9 mm, obconic to ellipsoid, medium to dark brown, testa rugose; hilum approximately ½ the length of the seed; embryotega semilateral; aril cream-colored, slightly translucent, thick.

##### Specimens seen

**. BRAZIL. Espírito Santo**: Fundão, Alto Piaba, cultivado na casa de epífitas do MBML, fl., 13 Sep 1989, W. Boone 1349 (MBML); A.P.A. do Goiapaba-açú, Piabas, propriedade de Albino Casimiro, fl., 8 Nov 2007, A.P. Fontana & K.A. Brahim 2827 (MBML, RB). Santa Teresa, Alto do Julião, propriedade de João Luiz Rodrigues de Souza, fl., 23 Feb 2007, A.P. Fontana & K.A. Brahim 2975 (MBML, RB); Cabeceira do 25 de Julho, Julião, fl., 10 Nov 2007, L. Kollmann et al. 11839 (MBML).

##### Etymology.

The epithet means “white-striped”, making reference to the thin and always present, white to silver stripe along the midvein of this species’ leaves.

##### Distribution and habitat.

*Siderasis albofasciata* is known exclusively from the municipalities of Santa Teresa and Fundão, state of Espírito Santo (Fig. 2). It occurs in the understory of evergreen forests, in shady areas with shallow and rocky soil, with great leaf-litter accumulation.

##### Phenology.

It blooms from November to February. This species was collected in fruit in April, when mature and immature capsules were seen.

##### Conservation status.

According to Pellegrini (2017), *S. albofasciata* should be considered as Critically Endangered [CR, B1ac(ii, iii, iv)+B2ab(ii, iii, iv)+C2a(i)].

##### Affinities.

*Siderasis albofasciata* is similar to *S. fuscata* due to its leaves being of a different color along the midvein of the adaxial side, abaxially vinaceous, and inflorescences covered with rusty hairs. However, *S. albofasciata* can be readily differentiated by its sessile to subpetiolate leaves covered by hyaline to light brown indumentum (vs. petiolate leaves with bright red to red indumentum, in *S. fuscata*), a well-defined white stripe along the midvein on the adaxial side of the blade (vs. sometimes blotched silver to metallic light green), main axis of the synflorescence elongate (vs. inconspicuous), bracteoles present (vs. bracteoles absent), cincinni (3–)5–8-flowered [vs. 1–3(–4)-flowered], anthers purple, filaments and style apically purple (vs. androecium and gynoecium completely white), testa brown and rugose (vs. grey to light grey and foveolate), and aril cream-colored, thick and slightly hyaline (vs. aril colorless and inconspicuous). It is also similar to *S. almeidae* and *S. medusoides* due to the leaf blades adaxially hispid, abaxially lanate, and presence of bracteoles in the cincinni. *Siderasis albofasciata* can be easily differentiated from all the accepted species in the genus by the peculiar coloration pattern in its androecium and gynoecium.

Furthermore, *S. albofasciata* produces unique axillary flagelliform-shoots after its flowering period. Each flagelliform-shoot is homologous to a daughter ramet, consisting of an extremely elongate stem, that may or not develop leaf blades (sometimes the blades are very reduced or absent), and a terminal rosette that roots after it touches the soil. This clonal propagation strategy gives this species a chandelier appearance, similar to many epiphytic bromeliads. This clonal propagation strategy is unique within subtribe Dichorisandrinae (Pellegrini 2017).

**Figure 6.**
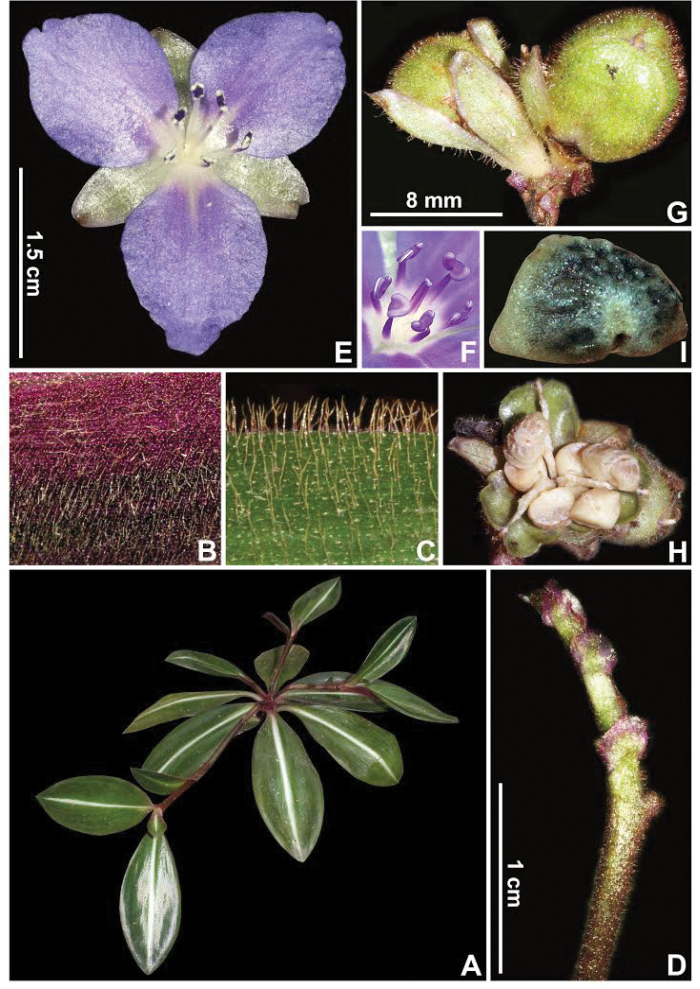
*Siderasis albofasciata* M.Pell. **A** habit, showing the well-defined white stripe along the midvein of the leaves and the flagelliform-shoots with terminal rosettes **B** detail of the abaxial side of the leaf, showing the light-brown lanate indumentum **C** detail of the adaxial side of the leaf, showing the light-brown hispid indumentum **D** detail of the inflorescence, showing the solitary cincinnus **E** flower **F** detail of the androecium and the gynoecium **G** detail of the capsules, the left one immature with evident accrescent sepals and the right one mature **H** detail of an open capsule, showing the biseriate to partially uniseriate, arillate seeds **I** dorsal view of a seed, showing the semi-lateral embryotega and the cream-colored, slightly translucent and thick aril. Photograph **F** by L. Kollmann, remaining by M.O.O. Pellegrini.

#### 2. Siderasis almeidae

Taxon classificationPlantaeCommelinalesCommelinaceae

M.Pell. & Faden sp. nov.

urn:lsid:ipni.org:names:77164153-1

Figs 2 , 7

##### Diagnosis.

Similar to *S. fuscata* due to its rusty indumentum in the leaves, lilac to purple rhomboid petals and white anthers. Also, similar to *S. albofasciata* due to its sessile to subpetiolate leaves, blades adaxially hispid and abaxially lanate, present bracteoles, and purple filaments and style. Nevertheless, *Siderasis almeidae* is peculiar in lacking terminal tubers in the roots, subterraneous stems, and having aerial stems elongate and trailing in the leaf litter, leaves entirely green, fleshy showy sepals, and a densely lanate ovary.

##### Type.

BRAZIL. Bahia: Itamarajú, Fazenda Pau Brasil, caminho para o Monte Pescoço, fl., 19 Nov 2015, M.O.O. Pellegrini & R.F. Almeida 493 (holotype: RB barcode RB01132619!; isotype: US!).

##### Description.

***Herbs*** ca. 20–45 cm tall, terrestrial. ***Roots*** thin, fibrous, terminal tubers absent. ***Rhizomes*** only covered by leaf litter. ***Subterraneous stems*** absent. ***Aerial stems*** trailing, only covered by leaf litter, unbranched to little branched, produced directly from the short rhizome; internodes elongate, green, sparsely lanate, becoming glabrous at age, hairs light brown to rusty; flagelliform-shoots (ramets) absent. ***Leaves*** spirally-alternate, forming a rosette at the apex of the stems, sessile to subpetiolate; sheaths 1.5–3.2 cm long, green, lanate, margin densely lanate, hairs light brown to rusty; subpetiole 0.8–4.6 cm long to inconspicuous, D-shaped in cross section, canaliculate, green, hispid, margin densely lanate, hairs light brown to rusty; blades 12.6–25.7 × 4–9.1 cm, succulent, elliptic or narrowly obovate to obovate, base cuneate, margins green, slightly revolute, densely lanate, apex acute, curved downwards, adaxially green to dark green, hispid, hairs light brown to rusty, abaxially light green, lanate, light brown to hairs rusty; midvein adaxially inconspicuous to conspicuous, slightly impressed, abaxially prominent, obtuse, secondary veins 6–8 pairs, adaxially conspicuous, slightly impressed, abaxially slightly prominent, becoming more evident adaxially when dry. ***Synflorescence*** composed of a solitary main florescence, or with 1–3(–5) coflorescences. ***Main florescence (inflorescence)*** reduced to a solitary pedunculate cincinnus; basal bract triangular, 2.4–4.6 × 1.1–2.2 cm, slightly cymbiform, amplexicaulous, green, hispid, hairs rusty, opaque at the base and margins; inflorescence main axis 2.2–8.6 mm long, green, densely hispid, hairs rusty; cincinni bract 1.1–3.6 × 0.4–1.4 cm, narrowly triangular, amplexicaulous, green, hispid, hairs rusty, apex acuminate; cincinni 5–11-flowered, peduncles 0.7–1.8 cm long, green, densely hispid, hairs rusty, reflexed in fruit; bracteoles 7.4–15.3 × 3.8–7.4 mm, broadly triangular, sessile, revolute, green at pre-anthesis, becoming purple at anthesis, hispid to densely hispid, hairs rusty, apex acuminate. ***Flowers*** bisexual, actinomorphic, 1.6–2.2 cm diameter, sessile; pedicel inconspicuous at anthesis, elongated in fruit, 0.8–2.2 mm long; floral buds 5.7–7.6 × 4.6–6.5 mm, broadly ellipsoid to broadly obovoid, green, apex obtuse to truncate; sepals 6.8–10.9 × 2.3–5.9 mm, ovate to broadly ovate, fleshy, externally green, densely hispid, hairs rusty, internally lilac to purple, glabrous, margins hyaline to hyaline lilac, apex acute; petals 8–18.2 × 6.4–8.1 mm, rhomboid to broadly obtrullate, purple to bluish purple, base cuneate, margin entire, rarely irregularly lacerated, apex obtuse to rounded; stamens equal, filaments 2.4–4.8 mm long, straight, lilac to purple, anthers 0.7–2.3 × 0.7–1.9 mm, anther sacs white, connectives quadrangular, white; ovary 1.8–2.9 × 1.2–2.1 mm, broadly oblongoid, white, densely lanate, hairs hyaline, style 3.6–4.8 mm long, straight, purple; stigma annular-truncate, purple, papillate. ***Capsules*** (immature) 5.7–6.8 × 5.9–7.2 mm, subglobose to broadly oblongoid in outline, smooth, green, when mature light brown, hispid, hairs rusty. ***Seeds*** unknown.

**Specimens seen (paratypes). BRAZIL. Bahia**: Itamarajú, ca. 2 km da Estrada BR-101 ao S de Itamarajú, fl., 5 Apr 1971, T.S. Santos 1559 (CEPEC, K); Fazenda Pau Brasil, ca. 5 km ao NW de Itamarajú, 17°1’ S 39°33’ W, fl., fr., 19 Sep 1978, S.A. Mori et al. 10730 (CEPEC, K, NY, RB, US); fl., 31 Oct 1979, L.A. Mattos Silva & H.S. Brito 692 (CEPEC, K, US). Prado, rod. BA-001, a 61 km ao N de Alcobaça, fl., 19 Mar 1978, S.A. Mori et al. 9739 (CEPEC, RB); km 21 of road from Itamarajú to Prado, forest on N side near logging operation, fl., 9 Feb 1993, J.A. Kallunki & J.R. Pirani 474 (NY, SPF).

##### Etymology.

The epithet honors Brazilian botanist Rafael Felipe de Almeida, a prominent specialist in Malpighiaceae, contributor in the studies of Commelinaceae, husband of the first author, and co-collector of the holotype, for his unmeasurable support in the field and in my research.

##### Distribution and habitat.

*Siderasis almeidae* is confined to the municipalities of Itamarajú and Prado, Bahia (Fig. 2). It occurs in the “mata higrófila” vegetation with emerging rocky formations, in shady and moist areas. In the type locality, the subpopulations were found growing in great accumulations of leaf litter, among dense clusters of Marantaceae. The area is greatly disturbed, and within private property.

##### Phenology.

It was found in bloom from September to April, beginning to fruit in September, but mature fruits are unknown.

##### Conservation status.

*Siderasis almeidae* has considerably narrow EOO (ca. 180.390 km^2^) and AOO (ca. 2800 km^2^). Most of the known collections were made in the type locality, in a small forest patch inside a private cattle farm. None of the known subpopulations is protected by a conservation unit, and the southern region of Bahia has few undisturbed areas of Atlantic Forest, being subjected to ongoing deforestation, cattle breeding, and several crops. The subpopulations of *S. almeidae* are small to medium-sized (with ca. 20 individuals), but mainly composed of clonal individuals. Thus, following the IUCN (2001) criteria, we suggest *S. almeidae* to be considered Critically Endangered [CR, A2abcd+B2ab(i, ii, iii, iv, v)+C1].

##### Affinities.

*Siderasis almeidae* is similar to *S. fuscata* due to their rusty indumentum covering the leaf blades, inflorescences and sepals, lilac to purple petals, and white anthers. It is also similar to *S. albofasciata* due to its sessile to subpetiolate leaves, present bracteoles, and purple filaments and style. Furthermore, *S. almeidae* is similar to *S. medusoides*, due to their sessile flowers, purple filaments and style, and white anthers.

**Figure 7.**
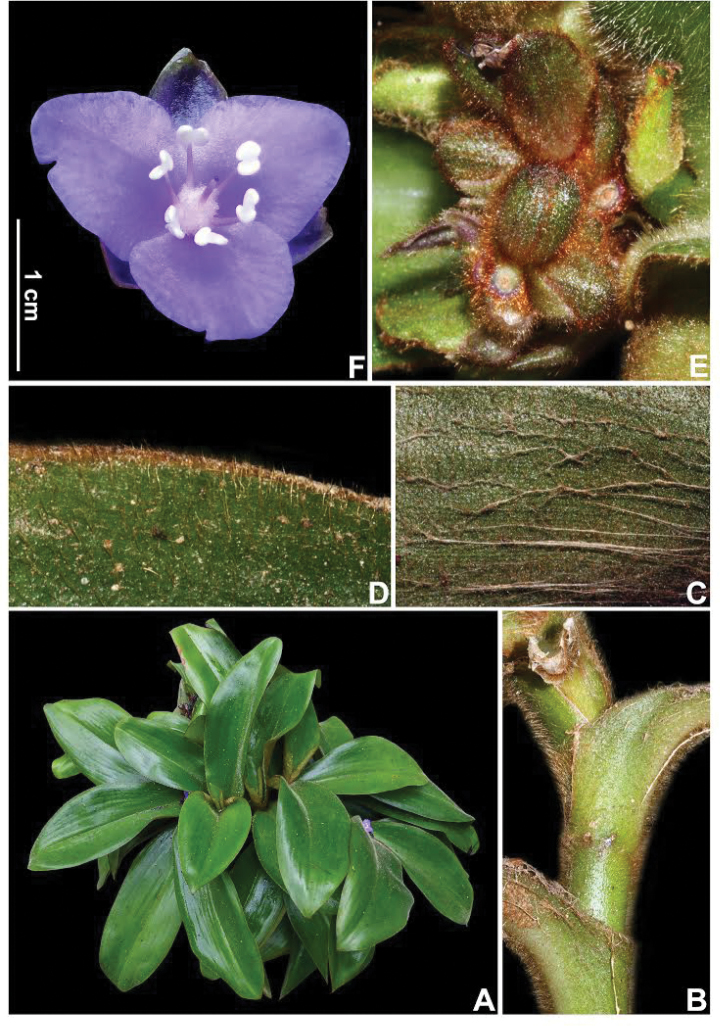
*Siderasis almeidae* M.Pell. & Faden. **A** habit, showing a fertile rosette **B** detail of the elongated aerial stem, showing the rusty internodes and leaf-sheaths **C** detail of the lanate indumentum on the abaxial side of the leaf blade **D** detail of the hispid indumentum on the adaxial side of the leaf blade **E** detail of the inflorescence, showing the contracted cincinnus and some floral buds **F** front view of a flower, showing the fleshy and internally purple sepals, and the lanate ovary. Photographs **A, F** by M.A.N. Coelho, remaining photographs by M.O.O. Pellegrini.

#### 3. Siderasis fuscata

Taxon classificationPlantaeCommelinalesCommelinaceae

(Lodd.) H.E.Moore, Baileya 4: 28. 1956.

Figs 1A , 2 , 8

Pyrrheima fuscatum (Lodd.) Backer, Handb. Fl. Java 3: 37. 1924.
Pyrrheima loddigesii var. minus (Hassk.) C.B.Clarke in Candolle & Candolle, Monogr. Phan. 3: 272. 1881.
Pyrrheima loddigesii Hassk., Flora 52: 367. 1869, nom. illeg.
Pyrrheima minus Hassk., Flora 52: 368. 1869. Holotype. BRAZIL. Rio de Janeiro. Rio de Janeiro, Corcovado, fl., s.dat., C. Gaudichaud 137 (P barcode P01799823!).
Tradescantia hirsutissima Pohl *ex* Seub., *in* Martius, Fl. bras. 3(1): 254. 1855, pro. syn.
Siderasis acaulis Raf., Fl. Tellur. 3: 67. 1837, nom. illeg.
Tradescantia fuscata Lodd., Bot. Cab. 4: t. 374. 1820. Lectotype (designated by Pellegrini 2017). [illustration] Original parchment plate of “The Botanical Cabinet” at the British Museum Library and later published in Loddiges, Bot. Cab. 4: t. 374. 1820. Epitype (designated by Pellegrini 2017). BRAZIL. Rio de Janeiro. Rio de Janeiro, Floresta da Tijuca, FEEMA, Parque Nacional da Tijuca, fl., fr., 7 Nov 2012, M.O.O. Pellegrini 217 (RB barcode RB01093071!).

##### Description.

***Herbs*** ca. 20–30 cm tall, terrestrial. ***Roots*** thin, fibrous, terminal tubers present. ***Rhizomes*** buried deep in the ground. ***Subterraneous stems*** with internodes elongate, brownish vinaceous to vinaceous with white spots, hirsute, hairs reddish brown. ***Aerial stems*** short to inconspicuous, unbranched; internodes weakly to moderately elongate, vinaceous with white spots, hirsute, hairs reddish brown; flagelliform-shoots (ramets) absent. ***Leaves*** spirally-alternate, forming a rosette at the apex of the aerial stems, petiolate; sheaths 1–1.5 cm long, hirsute, hairs dark red; petiole 2.7–9.6(–11.4) cm long, terete, canaliculate, C-shaped in cross section, succulent, green to dark green with dense vinaceous spots, spots sometimes covering almost all the petiole, hirsute, hairs red to dark red; blade (4.8–5.6–)7.8–21.1(–23.6) × (2–)3–9.6 cm, succulent, elliptic to obovate to broadly obovate, rarely lanceolate, base cuneate, margins green, flat, densely hirsute, apex acute to obtuse or rounded, slightly curved downwards, adaxially dark green, with a silver to light green, blotched silver to metallic light green along the midvein or not, abaxially light green, with dense vinaceous spots or not; midvein conspicuous, adaxially impressed, abaxially prominent, obtuse, secondary veins 3–6 pairs, adaxially conspicuous, impressed, abaxially inconspicuous, becoming more evident on both sides when dry. ***Synflorescence*** composed of a solitary main florescence, or with (1–)2–6(–7) coflorescences. ***Main florescence (inflorescence)*** reduced to a solitary pedunculate cincinnus; basal bract 1.5–2.2 × 0.3–1 cm, triangular, cymbiform, dorsally keeled, light pink, hirsute, hairs rusty to dark red, hyaline at the base and margins, apex acuminate; inflorescence main axis inconspicuous; cincinni bract 0.8–2 × 0.2–0.9 cm, triangular, slightly cymbiform, amplexicaulous, pink to vinaceous, hirsute along the midvein, hairs red, base hyaline, margins light brown to golden, glabrous, hyaline, apex acuminate; cincinni 1–3(–4)-flowered, peduncle 1.3–7.4 cm long, light brown, hirsute, hairs red, reflexed in fruit, more rarely also spirally-coiled in fruit; bracteoles absent. ***Flowers*** bisexual, actinomorphic, 2–2.8 cm diameter, pedicellate; pedicel 1–5.6 mm long, light brown, hirsute, hairs red, reflexed and elongate in fruit; floral buds 0.8–1.2 × 0.4–0.6 cm, ovoid, light brown to light pink, apex acuminate; sepals 0.8–1.4 × 0.3–0.8 cm, ovate to triangular, membranous, externally light brown, hirsute, hairs red, internally pink to vinaceous, glabrous, margin hyaline, apex acuminate; petals 1.2–1.6 × 1.2–1.5 cm, rhomboid to broadly obtrullate, rarely orbicular, pale lilac to lilac, proximal third gradually white, base cuneate, margin entire, sometimes irregularly lacerated, glabrous, apex obtuse to rounded, sometimes irregularly lacerated; stamens equal, filaments 3.5–6.3 mm long, straight, white, anthers 1.5–2.1 × 3–4.2 mm, anther sacs white, connectives quadrangular, white; ovary globose, 2.9–3.7 × 2.3–3.2 mm, white, densely hirsute, hairs white at base, gradually becoming rusty, then dark red terminally, style 3.1–4.4 mm long, straight, white; stigma annular-truncate, white, papillate. ***Capsules*** ellipsoid to fusiform in outline, 1.1–1.6 × 0.5–0.8 cm, smooth, light greenish brown with minute purple spots near the base and 3 longitudinal atro-vinaceous stripes along the septa, when mature light brown with 3 longitudinal black stripes along the septa, hirsute, hairs red. ***Seeds*** obconic to ellipsoid, dorsoventrally compressed, ventrally slightly ridged, 2.9–4.4 × 2.9–3.6 mm, grey to light grey, testa foveolate, ventral face slightly cleft on the side towards the embryotega; hilum longer than ½ the length of the seed; embryotega semilateral; aril hyaline, inconspicuous.

##### Specimens examined.

**BRAZIL. Rio de Janeiro**: Niterói, Itaipu, P.E. Serra da Tiririca, Alto Mourão, fl., 15 Jan 1982, V.F. Ferreira et al. 2104 (RB); divisa entre os municípios de Niterói e Maricá, entre Itacoatiara e Itaipuaçu, Alto Mourão, fl., 11 Sep 2007, A.A.M. Barros & M. Pontes 3127 (RFFP). Rio de Janeiro, s.loc., fl., s.dat., Mr. Boag s.n. (K barcode K001190685); s.loc., fl., s.dat., Mrs. Graham s.n. (K barcode K001190684); s.loc., fl., 1816–1821, A. Saint-Hilaire A/683 (P); s.loc., fl., fr., 1832, Riedel s.n. (P barcodes P01730357, P01730358); Corcovado, fl., fr., 1831–1833, C. Gaudichaud 337 (P 3 ex); fl., Jul 1837, G. Gardner 847 (K barcode K001190683); Cova da Onça, fl., 15 Aug 1861, A.M. Glaziou 527 (NY, P); fl., 17 Aug 1869, A.M. Glaziou 4285 (P 2 ex); fl., Jul 1878, J. Miers 3534 (K, P); fl., fr., 5 Dec 1889, P. Schwacke 6699 (RB); fl., 5 May 1892, A. Ducke s.n. (RB 64); Tijuca, rio Trapicheiros (Fábrica da Cheetos), fl., Nov 1925, J.S. Kuhlmann s.n. (RB 19282, U barcode U1210766); fl., fr., 4 Mar 1943, A.P. Duarte & C.T. Rizzini 8 (RB); fl., 6 Nov 1944, P. Occhioni 50 (RB); Parque Natural da Tijuca, Matas do Pai Ricardo, fl., 29 Oct 1975, D.S. Araújo et al. 883 (GUA); fl., 30 Oct 2013, M.O.O. Pellegrini 404 (RB); fl., 15 Nov 2013, L.S.B. Calazans & R.T. Valadares 234 (RB); road to Vista Chinesa, next to the Biological Station, fl., 18 Aug 1960, C. Angeli 230 (GUA); Setor das Paineiras, next to Pedra do Beijo, fl., 15 Nov 1965, J.P.P. Carauta 286 (GUA); road to Vista Chinesa, fl., fr., 31 Oct 1969, J.P.P. Carauta 923 (GUA); Santa Cruz, fl., 6 Jul 1972, E. Lagasa s.n. (HB 71875); Pedra da Gávea, fl., 13 Jul 1966, D. Sucre 1304c (HB, RB); Alto da Boa Vista, Morro Queimado, next to the FEEMA building, fl., 26 Oct 2000, F. Pinheiro et al. 557 (HB); Estrada da Guanabara, Parque Lage, 25 Jan 1968, fl., D. Sucre 2161 (RB); Reserva Florestal do Jardim Botânico, fl., 19 Jan 1969, D. Sucre & P.J.J. Braga 4472 (RB); fl., 22 Dec 1971, D. Sucre 8152 (RB); Matas da Lagoinha, fl., 18 September 1946, P. Occhioni 692 (RB); fl., 6 Mar 1978, V.F. Ferreira et al. 256 (RB); fl., 11 Nov 1946, P. Occhioni 781 (RFA); brook trail between Paineiras and Jardim Botânico, fl., 4 Dec 1928, L.B. Smith s.n. (US barcode US1540545).

##### Specimens examined (cultivated).

**ENGLAND. Greater London**: London, Royal Botanic Gardens, Kew, cultivated at the Nepenthes House, Kew, fl., 1908, s. leg. s.n. (K); fl., fr., Jun 1879, N.L. Brown s.n. (K); fl., 1967, Mason 458/61 (K); fl., 9 Jul 1974, Jodrell Laboratory s.n. (K 458-61-45801).

##### Etymology.

The epithet “*fuscata*” means dark-colored, in allusion to the red to bright red hairs that cover almost the entire plant, in opposition to the normally hyaline hairs in most Commelinaceae.

##### Distribution and habitat.

*Siderasis fuscata* is endemic to the municipalities of Rio de Janeiro (with several localities inside Floresta da Tijuca) and Niterói (with just one locality, Alto Mourão), in the Rio de Janeiro state (Fig. 2). It occurs in the vegetation on hillsides (mata de encosta) near the littoral, in shady areas with shallow and rocky soil.

##### Phenology.

It blooms from August to May and fruits from January to May, although fructification seems to be an uncommon event since few fruiting specimens were seen or collected.

##### Common name.

“*violeta-silvestre*”, “*orelha-de-urso*”, “*pelo-de-urso*”, “*trapoeraba-peluda*”, “brown spiderwort”, “bear ears”.

##### Conservation status.

*Siderasis fuscata* is one of the few Commelinaceae included in the *Lista da Flora Brasileira Ameaçada de Extinção* (List of the Threatened Brazilian Flora; Fundação Biodiversitas 2009) and in the *Lista Oficial das Espécies da Flora Brasileira Ameaçadas de Extinção* (Official List of the Threatened Species of the Brazilian Flora; MMA 2008), at both lists classified as Data Deficient (DD). In the recently published Commelinaceae chapter of the *Livro Vermelho da Flora do Brasil* (Red Book of the Brazilian Flora; Aona-Pinheiro et al. 2013), *S. fuscata* is classified as Endangered (EN) by the authors, based on existing published data.

The subpopulation from Niterói is disjunct from the others in Rio de Janeiro, due to the urban area of both cities. It possesses a considerably small EOO (ca. 7000 km²), with the population being severely fragmented. Despite all the extant subpopulations being inside conservation units (i.e. Parque Nacional da Tijuca and Parque Estadual Serra da Tiririca), they are considerably small, composed mainly of clonal individuals, with no more than 30 mature individuals. Only a small number of fertile individuals can be found during the flowering season in each population, and very few fruits are produced. All these areas are extremely susceptible to real-estate development, deforestation, and have many invasive species, with areas like Parque Estadual Serra da Tiririca being especially affected by human-related forest fires. The subpopulations from Pedra da Gávea and Corcovado are probably extinct, or nearly so, since no recent collection in either areas is known by the authors. A total of 250 mature individuals is estimated for the overall population, based on our field observations. Added to the above factors, *S. fuscata* is appreciated as an ornamental plant all over the world due to its exotic foliage and beautiful flowers, so the few known extant subpopulations are also a target of illegal collection for exotic plant growers from all over the world. Thus, following the IUCN criteria (2001), we suggest *S. fuscata* be considered Critically Endangered [CR, A2abcde+B1ab(i,ii,iii,iv,v)+B2a(i, ii)+ C2a(i)+D2].

##### Affinities.

*Siderasis fuscata* is similar to *S. albofasciata* in their variegated leaf blades, and similar to *S. almeidae* and *S. medusoides* in their white anthers. Nevertheless, it can be readily distinguished from all species of *Siderasis* by its petiolate leaves, red to bright red indumentum covering almost the entire plant (*vs.* sessile to subsessile leaves, light brown to hyaline indumentum), cincinni without bracteoles (*vs.* bracteoles present), acuminate flower buds and sepals (*vs.* obtuse to rounded), androecium and gynoecium completely white (*vs.* androecium and gynoecium partially bluish, lilac or purple), ovary and capsules hirsute (*vs.* velutine, hispid or lanate), seeds with light grey to grey and foveolate testa (*vs.* medium to dark brown and rugose or scrobiculate testa), and hyaline and inconspicuous aril (*vs.* aril cream-colored, slightly translucent and thick).

**Figure 8.**
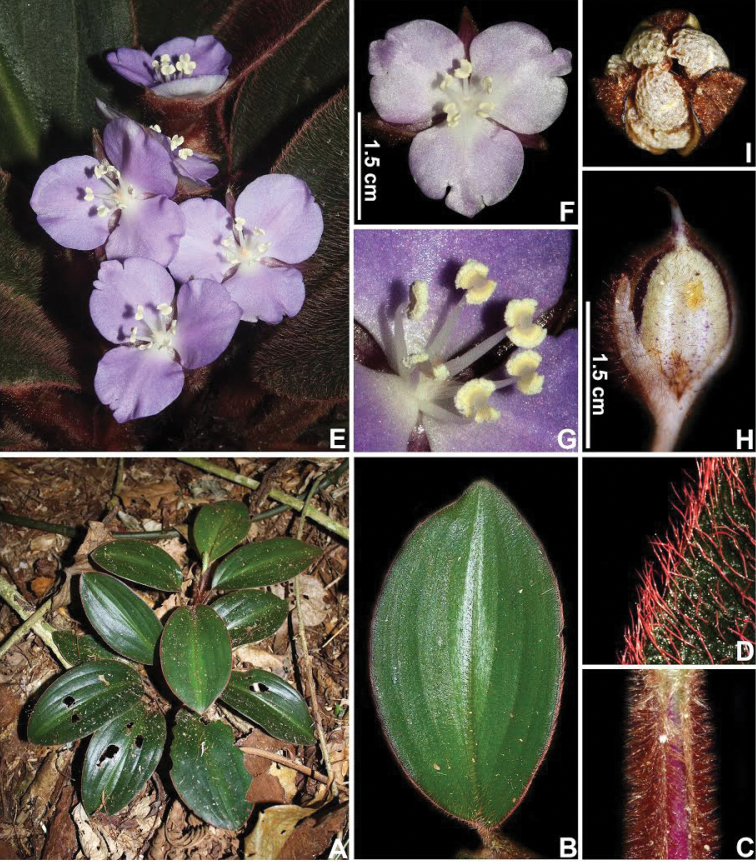
*Siderasis fuscata* (Lodd.) H.E.Moore. **A** habit, showing a mature rosette and green leaves **B** detail of leaf with a silvery blotch along the midvein **C** detail of the canaliculate petiole, showing the rusty hairs **D** detail of the bright-red hirsute indumentum **E** upper view of a fertile rosette, showing many lilac flowers open at the same time **F** front view of a pale-lilac flower, showing the lacerated petals and completely white androecium and gynoecium **G** detail of the androecium and the gynoecium **H** detail of a mature capsule, showing the atro-vinaceous longitudinal stripes **I** detail of an open capsule, showing the biseriate to partially uniseriate, inconspicuously arillate seeds, with grey foveolate testa. Photographs by M.O.O. Pellegrini.

#### 4. Siderasis medusoides

Taxon classificationPlantaeCommelinalesCommelinaceae

M.Pell. & Faden sp. nov.

urn:lsid:ipni.org:names:77164154-1

Figs 2 , 9

##### Diagnosis.

Similar to *S. almeidae* due to its sessile to subpetiolate, entirely green leaves, present bracteoles, sessile flowers, purple filaments and style combined with white anthers, and oblongoid to broadly oblongoid capsules. *Siderasis medusoides* is distinct due to its membranous leaves, elongate and tangled cincinni, small flowers, and purple to dark blue and elliptic to narrowly obovate or spatulate petals.

##### Type.

BRAZIL. Espírito Santo: Marilândia, perímetro urbano, na Estrada para São Pedro, fragment de floresta junto a uma serraria de madeira, a ca. 100 m do portão da serraria, em encosta de morro, 19°24'30.5"S 40°32'1.8"W, fl., fr., 20 Jan 2011, P. Fiaschi et al. 3489 (holotype: SPF barcode SPF200900!; isotype: MBML barcode MBML42135!).

##### Description.

***Herbs*** ca. 5–10 cm tall, rhizomatous, terrestrial. ***Roots*** with terminal tubers present. ***Rhizomes*** shallowly buried in the ground. ***Subterraneous stems*** short to inconspicuous, unbranched, dark green to vinaceous to brown, sparsely lanate, hairs light brown to hyaline. ***Aerial stems*** short to inconspicuous, unbranched; internodes inconspicuous to weakly elongate, green, lanate, hairs light brown to hyaline; flagelliform-shoots (ramets) absent. ***Leaves*** spirally-alternate, forming a rosette; sheaths 0.8–1.4 cm long, hispid, hairs hyaline to light brown; subpetiole 0.4–4.6 cm long to inconspicuous, D-shaped in cross section, canaliculate, dark green to vinaceous, hispid, hairs light brown to hyaline; blades 10–24.4 × 5.9–11.2 cm, elliptic to broadly elliptic, membranous, adaxially dark green, hispid, hairs light brown to hyaline, abaxially green to vinaceous, hispid to lanate, hairs light brown to hyaline, base cuneate, margins green, revolute, lanate, hairs light brown to hyaline, apex obtuse, rarely acute, straight; midvein conspicuous, adaxially impressed, abaxially prominent, acute, secondary veins 2–7 pairs, inconspicuous on both sides, becoming more conspicuous on both sides when dry. ***Synflorescence*** composed of a solitary main florescence, or with 1–15 coflorescences. ***Main florescence (inflorescence)*** reduced to a solitary pedunculate cincinnus; basal bract 7.6–10.4 × 4.6–6.2 cm, broadly elliptic to broadly ovate, slightly cymbiform, amplexicaulous, green, sparsely hispid, hairs rusty, opaque at the base and margins; inflorescence main axis 2.3–4.8 cm long, green, densely hispid, hairs rusty; cincinni bract ovate, amplexicaulous, 2.4–4.9 × 1.5–4 mm, green, hispid, hairs rusty, apex acute; cincinni (5–)8–26-flowered, peduncles 5.6–12.7 mm long, green, densely hispid, hairs rusty, reflexed in fruit; bracteoles 0.9–1.5 × 0.8–1.3 mm, broadly triangular, sessile, flat, green, hispid, hairs rusty, apex obtuse. ***Flowers*** bisexual, actinomorphic, 0.9–1.2 cm diameter, sessile; pedicel inconspicuous, elongate in fruit, 1–2.2 mm long, green, hispid, hairs light brown to rusty; floral buds 2.6–5.4 × 2–3.7 mm, broadly ellipsoid to broadly obovoid, light green, apex obtuse to truncate; sepals 3.7–6.7 × 2.2–3.6 mm, elliptic to obovate, the uppermost external and broader than the others, membranous, externally light green to green, sparsely hispid, hairs light brown to rusty, internally light green, purple towards the apex, glabrous, margin hyaline, apex obtuse; petals 4.4–10.1 × 1.9–6.7 mm, elliptic to narrowly obovate to spatulate, the lowermost usually broader than the others, bluish purple to dark blue, margin entire to irregularly lacerated, apex obtuse to round, irregularly lacerated; stamens 6, equal, filaments 2.6–3.4 mm long, bluish purple to dark purple, anthers 0.8–1 × 1–1.3 mm, anther sacs semicircular, divergent, white, connectives quadrangular, white; ovary broadly oblongoid, 1.2–1.9 × 1–1.5 mm, white, densely hispid, hairs white; style 1.3–4.7 mm long, straight, bluish purple to dark blue, lilac at the terminal end; stigma annular-truncate, lilac to white, papillate. ***Capsules*** 6.8–9.4 × 6.7–7.8 mm, oblongoid to broadly oblongoid, smooth, green, hispid, hairs rusty. ***Seeds*** 3.6–4.1 × 2.6–3.2 mm, obconic to ellipsoid, medium to dark brown, testa rugose; hilum longer than ½ the length of the seed; embryotega semilateral; aril cream-colored, slightly translucent, thick.

##### Specimens seen (paratypes).

**BRAZIL. Espírito Santo**: Marilândia, rodovia Marilândia-Rio Bananal, ca. 1 km N de Marilândia, remanescente de floresta junto a Cerâmica Floresta, fl., 6 Dec 1994, J.R. Pirani et al. 3421 (NY, SPF); Liberdade, propriedade de Deoclécio Lorenccini, 19°21’ 7” S 40°30’ 51” W, fl., fr., 22 Mar 2007, V. Demuner et al. 3429 (HERB, MBML); propriedade de Sônia e Reinaldo Bautz, 19°20’ 8” S 40°32’ 8” W, fl., 10 Dec 2007, V. Demuner et al. 4682 (MBML). Santa Leopoldina, Colina Verde (Morro do Agudo), propriedade de Israel Elias Ramos, trilha da casa, 20°6’ 12” S 40°26’ 34” W, fl fr., 13 Mar 2007, V. Demuner et al. 3118 (MBML).

##### Etymology.

The epithet alludes to the extremely elongated cincinni, common in mature individuals of this species, due to their resemblance to the snakes that composed the hair of Medusa, one of the three Gorgon sisters from Greek mythology.

##### Distribution and habitat.

*Siderasis medusoides* is known from the municipalities of Marilândia and Santa Leopoldina, in the state of Espírito Santo (Fig. 2). It grows in lowland Atlantic Forest, in shady and moist areas with great leaf litter accumulation, 90–550 m above the sea level.

##### Phenology.

It blooms from December to March and fruits between January and March.

##### Conservation status.

*Siderasis medusoides* possesses narrow EOO (ca. 11037 km^2^) and AOO (ca. 2000 km^2^), and based solely on distribution data should be treated as Endangered (EN). Nonetheless, it is known from only five collections in three different localities. They were made within urban areas, and these localities have suffered greatly with direct anthropic influence and deforestation in recent years. We have made several attempts to recollect *S. medusoides*, but they were all unsuccessful. Thus, we suggest that *S. medusoides* be considered Critically Endangered [CR, A2abcd+B2ab(i, ii, iii, iv)+C1+C2b+D2].

##### Affinities.

*Siderasis medusoides* is similar to *S. almeidae* and *S. albofasciata*, due to their sessile to subpetiolate leaves, inflorescence with elongate main axis, bracteolate cincinni, sessile flowers, and purple filaments and style combined with white anthers. Nevertheless, it can be easily differentiated from *S. almeidae* by its inconspicuous subterraneous and aerial stems (*vs.* subterraneous stems absent and aerial stems elongate, in *S. almeidae*), membranous leaves appressed against the soil (*vs.* succulent and ascending), membranous and internally light green sepals (*vs.* fleshy and internally lilac to purple), narrowly obovate to spatulate petals (*vs.* rhomboid to broadly obtrullate), and hispid ovary (*vs.* lanate). It can be differentiated from *S. albofasciata* by lacking flagelliform-shoots (*vs.* flagelliform-shoots produced after the fertile period, in *S. albofasciata*), concolorous and membranous leaves (*vs.* adaxially variegated, abaxially vinaceous, succulent leaves), petals entirely purple to bluish purple (*vs.* petals with white basal third), and white anthers (*vs.* anthers purple to bluish purple). *Siderasis medusoides* is peculiar due to its membranous leaves appressed to the soil, tangled and elongate cincinni, small flowers, and narrow petals.

**Figure 9.**
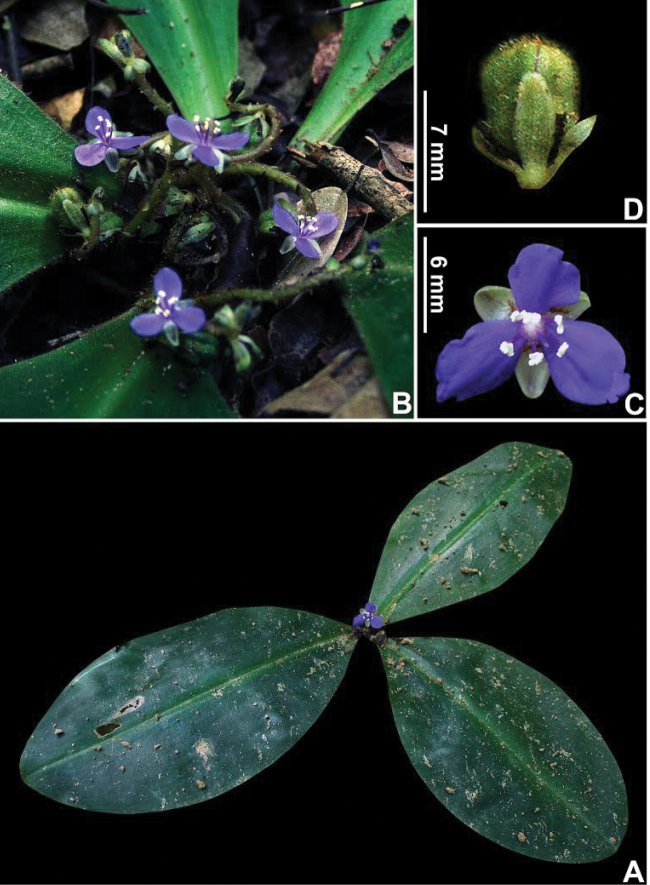
*Siderasis medusoides* M.Pell. & Faden. **A** habit, showing a fertile rosette **B** detail of the synflorescence, showing the elongated and tangled cincinni **C** front view of a flower, showing small ants near the flower center **D** detail of the capsule. Photographs by P. Fiaschi.

#### 5. Siderasis spectabilis

Taxon classificationPlantaeCommelinalesCommelinaceae

M.Pell. & Faden sp. nov.

urn:lsid:ipni.org:names:77164155-1

Figs 2 , 10 , 11

##### Diagnosis.

Very distinctive due to its vining habit, distichously-alternate leaves, blades asymmetric at base, main florescence a many-branched thyrse, with alternate cincinni, flowers zygomorphic, bisexual or staminate, stamens unequal, curved upwards, sigmoid filaments, and capsules globose and shallowly foveolate. It can be differentiated from *S. zorzanellii* by its membranous and velutine leaves, inflorescences always terminal in the secondary branches, petals dark mauve to vinaceous, rarely light pink or white, with margins ciliate with non-moniliform hairs.

##### Type.

Brazil. Rio de Janeiro: Santa Maria Madalena, morro atrás do Horto Santos Lima (sede do Parque Estadual do Desengano), fl., 19 Jan 1957, L.E. Mello-Filho 1172 (holotype: R barcode R000103716!; isotypes: RB!, SPF!, US!).

##### Description.

***Vines*** ca. 0.5–3 m tall, terrestrial. ***Roots*** unknown. ***Rhizomes*** unknown. ***Subterraneous stems*** unknown. ***Aerial stems*** twining, primary stem indefinite, densely branched, internodes elongate, 2.2–6.5 cm long, green, minutely velutine on both sides, hairs hyaline to light brown; secondary branches definite, unbranched, ca. 17–25 cm long, internodes elongate, 1.1–2.3 cm long, green, minutely velutine on both sides, hairs hyaline to light brown. ***Leaves*** distichously-alternate, evenly distributed along the secondary branches, sessile; sheaths 0.7–2 cm long, green to vinaceous, minutely velutine, with a line of eglandular hairs opposite the leaf above, margins setose, hairs hyaline to light brown; subpetiole 1.1–3.3 mm long to inconspicuous, C-shaped in section, canaliculate, membranous, green to dark green, minutely velutine on both sides, hairs hyaline to light brown; blades 4.6–11.8 × 1.6–2.5 cm, linear elliptic or linear lanceolate or linear oblong, membranous, adaxially dark green to green, becoming dark brown when dry, abaxially light green to green, becoming greyish green to olive-green when dry, minutely velutine on both sides, hairs hyaline to light brown, base slightly asymmetric to asymmetric, cuneate to narrowly rounded, margins vinaceous, flat, minutely velutine, hairs hyaline to light brown, apex acuminate to caudate, straight; midvein conspicuous, impressed adaxially, prominent, obtuse abaxially, secondary veins (3–)4–5 pairs, slightly conspicuous on both sides, becoming more evident when dry. ***Synflorescence*** composed of a solitary main florescence. ***Main florescence (inflorescence)*** a pedunculate, many-branched thyrse, with alternate cincinni, terminal in the secondary branches; basal bract leaf-like, amplexicaulous to sheathing, sheaths 1.2–4.8 mm long, minutely velutine, margins of the sheaths densely setose, blades 3.9–6.7 × 0.5–1.1 cm, green to dark green, minutely velutine on both sides, base opaque, margins minutely velutine, apex acuminate to caudate, hairs hyaline to light brown; peduncle 1–1.3 cm long, green, minutely velutine, hairs hyaline to light brown; cincinni bract 3.2–10.6 × 0.8–1.2 mm, linear triangular, mauve to vinaceous, minutely velutine on both sides, base truncate, margin sparsely setose, apex acuminate to caudate, hairs hyaline to light brown; cincinni 14–17 per thyrse, 3–8-flowered, peduncles 1.4–7.2 mm long, light green to pink, minutely velutine, hairs hyaline to light brown, erect in fruit; bracteoles 1.8–2.2 × 0.8–1.2 mm, ovate to broadly ovate, flat, cream-colored densely covered with vinaceous to pinkish purple spots to completely mauve to vinaceous, minutely velutine on both sides or only along the midvein, base rounded, margin hyaline, sparsely ciliate, apex hyaline, acute to obtuse, hairs hyaline to light brown. ***Flowers*** bisexual or staminate, zygomorphic, 1–1.3 cm diameter, pedicellate; pedicel 0.5–0.7 mm long, medium to dark mauve, sparsely minutely velutine, hairs hyaline to light brown, patent and slightly elongate in fruit; floral buds 3.5–4.4 × 2.4–3.8 mm, broadly ellipsoid to broadly obovoid, vinaceous to dark vinaceous, apex truncate; sepals 4.8–5.2 × 2–2.6 mm, narrowly ovate to elliptic, cymbiform, unequal, the uppermost external, broader and shorter than the others, fleshy, vinaceous to dark vinaceous, externally sparsely minutely velutine, hairs hyaline to light brown, internally glabrous, margin hyaline, glabrous to sparsely minutely velutine, hairs hyaline, apex obtuse, slightly purple; petals 5.1–6.3 × 2.8–3.6 mm, trullate to obovate, the lowermost narrower than the others, dark mauve to vinaceous, rarely light pink or white, base cuneate, margin entire, ciliate with dark mauve, eglandular, non-moniliform, uniseriate hairs, apex obtuse to rounded; stamens 6, unequal, the anterior longer than the posterior ones, curved upwards, filaments 1.8–4.6 mm long, sigmoid, white, terminal third dark mauve, anthers 1.2–1.4 × 0.8–1 mm, anther sacs dark mauve, connectives quadrangular in the shorter stamens and rectangular in the longer, dark mauve to purple; ovary 1.7–1.9 × 1–1.4 mm, ellipsoid to broadly ellipsoid, white, velutine, hairs hyaline, style 3.2–4 mm long, curved upward at the apex, white to pink, terminal third dark mauve; stigma annular-capitate, mauve to pink, papillate. ***Capsules*** and ***Seeds*** unknown.

##### Specimens seen (paratypes).

**BRAZIL. Rio de Janeiro**: Santa Maria Madalena, morro atrás do Horto Santos Lima (sede do Parque Estadual do Desengano), fl., 19 Jan 1957, L.E. Mello-Filho 1162 (R, RB, US); fl., 19 Jan 1957, L.E. Mello-Filho 1171 (R, RB, US).

##### Etymology.

The epithet means “admirable, remarkable, spectacular”, in allusion to its distinctive growth form, small flowers with a peculiar coloration, and the unique petal margins ciliate with non-moniliform hairs.

##### Distribution and habitat.

*Siderasis spectabilis* is confined to the type locality, in the native vegetation of the Horto Santos Lima (currently the headquarters of the Desengano State Park), in Santa Maria Madalena, state of Rio de Janeiro (Fig. 2). Nothing is known about this species habitat, since the original labels give no information on the area and all field expeditions to recollect this plant have been unsuccessful.

##### Phenology.

Since all known collections were done on the same day, *S. spectabilis* is only known to bloom during January. Fruits and seeds are unknown for this species.

##### Conservation status.

Due to the complete lack of information on the distribution, ecology and lack of any collections aside from the type specimens, according to the criteria proposed by IUCN (2001), *S. spectabilis* should be considered Data Deficient (DD), until new collections and data become available.

##### Affinities.

*Siderasis spectabilis* is morphologically closely related to *S. zorzanellii*, but *S. spectabilis* can be easily differentiated due to its inflorescences being always terminal in the secondary branches (*vs.* axillary in the primary branches or terminal in the secondary branches, in *S. zorzanellii*), and petals dark mauve to vinaceous, rarely light pink or white, and margins ciliate with non-moniliform hairs (*vs.* white and glabrous margins). All studied specimens were in excellent condition, and color of most organs could be easily described. Regarding color pattern in the androecium and gynoecium, *S. spectabilis* is similar to *S. albofasciata*. These are the only two species in the genus to present the upper third of filaments and style, and the anthers in the same color as the petals, contrasting greatly with the white base of filaments and style, and the white ovary of other species. Nevertheless, both species can be easily differentiated using vegetative or reproductive characters. One specimen (*L.E. Mello-Filho 1171*) is peculiar in being the only specimen with light-colored flowers. In the label, it is described by the collector as possessing white flowers. Nonetheless, while analyzing the duplicates available at R, RB, SPF and US, we noticed that a few flowers possessed pale pink pigment (particularly noticeable in the petals and stamens). We believe that these specimens might represent albino or semialbino individuals, and thus merit no taxonomic status, especially since they were collected at the same place and date as the remaining dark-flowered specimens.

Aona (2008), in her unpublished Ph.D. thesis, lists one of the paratypes of *S. spectabilis* under *Dichorisandra incurva* Mart. This is justified by her due to the specimens climbing habit, decumbent apical branches, distichously-alternate and sessile leaves, inflorescence composed of a pedunculate, many-branched thyrse, with alternate cincinni, and “white” [sic] flowers. Nevertheless, *S. spectabilis* can be easily differentiated by its erect inflorescences (*vs.* pendant to curved downwards, hence the name, in *D. incurva*), flower buds broadly ellipsoid to broadly obovoid, with truncate apex (*vs.* ellipsoid, with acute apex), sepals fleshy (*vs.* membranous), petals dark mauve to vinaceous, rarely light pink or white, with margins ciliate with non-moniliform hairs (*vs.* white with glabrous margins), stamens 6, anthers dorsifixed, 3 to 4 times shorter than the filaments, dehiscent by extrorse slits, and anther sacs divergent, semicircular, and expanded connectives (*vs.* stamens 6 or 5 + the upper one modified into a staminode, anthers basifixed, 3 to 4 times longer than the filaments, dehiscent by introrse slits, but functionally poricidal, anthers sacs parallel, elongate, and inconspicuous connectives). All these floral characters can be easily observed with the dissection of mature flower buds in herbarium specimens. The floral morphology of *D. incurva* is illustrated in Fig. 1I.

**Figure 10.**
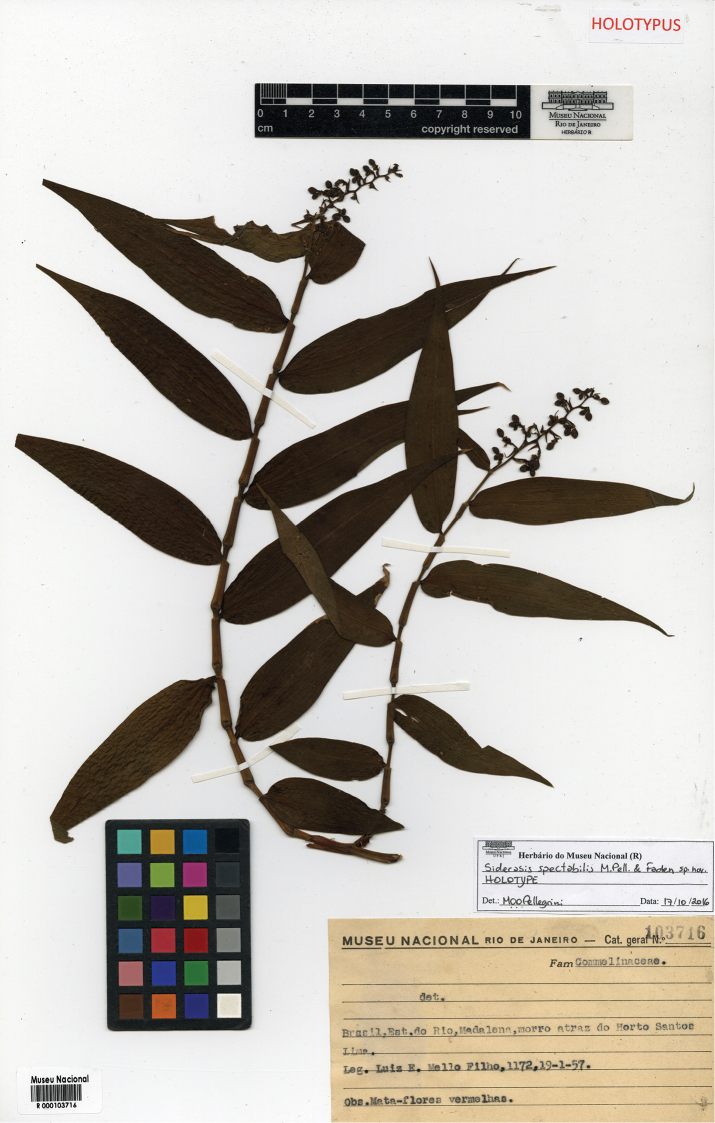
Holotype of *Siderasis spectabilis* M.Pell. & Faden. Image courtesy of the Museu Nacional, Rio de Janeiro, Brazil.

**Figure 11.**
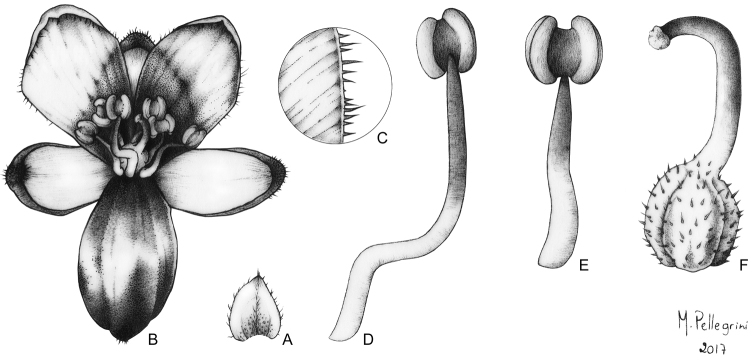
Line drawings of *Siderasis spectabilis* M.Pell. & Faden. **A** bracteole **B** front view of a bisexual flower, showing the petals ciliate with non-moniliform hairs **C** detail of the petal margin, showing the non-moniliform hairs **D** lower stamen, showing the rectangular anther connective **E** upper stamen, showing the quadrangular anther connective **F** Detail of the gynoecium, showing the velutine ovary and bent style. Line drawings by M.O.O. Pellegrini.

#### 6. Siderasis zorzanellii

Taxon classificationPlantaeCommelinalesCommelinaceae

M.Pell. & Faden sp. nov.

urn:lsid:ipni.org:names:77164156-1

**Figure 12.**
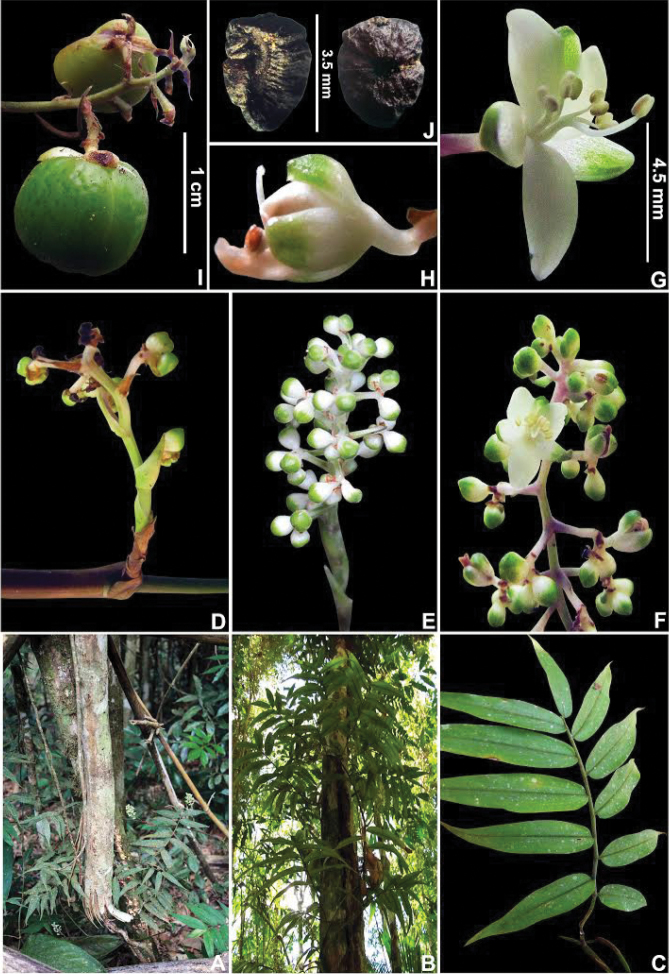
*Siderasis zorzanellii* M.Pell. & Faden. **A** habit, showing an immature individual **B** habit, showing a mature individual spirally ascending a tree **C** detail of a secondary branch, showing distichously-alternate, asymmetric leaves **D** detail of an axillary inflorescence, in the primary branch **E** detail of a terminal inflorescence, at pre-anthesis, in a secondary branch **F** detail of a terminal inflorescence, at anthesis, in a secondary branch, showing an open male flower **G** side view of a male flower, showing the unequal and sigmoid stamens **H** side view of a post-anthesis bisexual flower, showing the bent style **I** detail of the inflorescence bearing two mature capsules **J** dorsal and ventral view of the seeds, showing the rugose testa, cleft towards the semidorsal embryotega, and the C-shaped hilum. Photographs by J.P.F. Zorzanelli.

Figs 1A , 2 , 3B–C , 12

##### Diagnosis.

Similar to *S. spectabilis* due to its vining habit, distichously-alternate leaves, blades asymmetric at base, main florescence a many-branched thyrse, with alternate cincinni, flowers bisexual or staminate, zygomorphic, stamens unequal, curved upwards and sigmoid filaments. It can be differentiated from by its chartaceous and sparsely velutine leaves, inflorescences axillary in the primary branches or terminal in the secondary branches, and petals white with glabrous margins.

##### Type.

Brazil. Espírito Santo: Iúna, Serra do Valentim, ao lado do transecto 1, 20.4989°S, 41.4725°W, fl., 27 Mar 2014, J.P.F. Zorzanelli 969 (holotype: RB!; isotype: VIES!).

##### Description.

***Vines*** ca. 0.5–3 m tall, terrestrial. ***Roots*** thin, fibrous, terminal tubers present, fusiform. ***Rhizomes*** buried deep in the ground. ***Subterraneous stems*** inconspicuous. ***Aerial stems*** twining, primary stem indefinite, densely branched, internodes elongate, 4.3–10.6 cm long, green, minutely velutine on both sides, hairs hyaline to light brown; secondary branches definite, unbranched, (6.4–8–)15–34 cm long, internodes elongate, 2–2.3 cm long, green, minutely velutine on both sides, hairs hyaline to light brown. ***Leaves*** distichously-alternate, evenly distributed along the secondary branches, sessile; sheaths 2–2.7 cm long, green to brown, minutely velutine, with a line of eglandular hairs opposite the leaf above, margins setose to densely setose, hairs hyaline to light brown; subpetiole 2.9–3.5 mm long to inconspicuous, C-shaped in section, canaliculate, membranous, green to dark green, minutely velutine on both sides, hairs hyaline to light brown; blades 5.1–12.7 × 1.1–2.8 cm, linear elliptic to linear lanceolate, chartaceous, adaxially dark green to green, becoming dark brown when dry, abaxially light green to green, becoming greyish green to olive-green when dry, sparsely minutely velutine on both sides, hairs hyaline to light brown, base slightly asymmetric to asymmetric, cuneate to narrowly rounded, margins green to vinaceous, flat, glabrous, apex acuminate to caudate, straight; midvein conspicuous, impressed adaxially, prominent, obtuse abaxially, secondary veins 2–3 pairs, inconspicuous on both sides, becoming more evident when dry. ***Synflorescence*** composed of a solitary main florescence. ***Main florescence (inflorescence)*** a pedunculate, many-branched thyrse, with alternate cincinni, axillary in the primary branches or terminal in the secondary branches; basal bract reduced, rarely leaf-like, sessile, 1.7–2 × 0.2–0.4 cm, green, minutely velutine on both sides, base opaque, margins minutely velutine, apex caudate, hairs hyaline; peduncle 0.9–1.2 cm long, light green to green, minutely velutine hairs hyaline; cincinni bract linear triangular, 3–15.3 × 1.4–1.8 mm, green to brown, minutely velutine on both sides, base truncate, margin velutine, setose only at base, apex acuminate to caudate, hairs hyaline; cincinni 14–19 per thyrse, (1–)2–5-flowered, peduncles 1.2–5.3 mm long, white to pink, minutely velutine, hairs hyaline, erect in fruit; bracteoles ovate to broadly ovate, flat, 1–1.7 × 0.8–1.3 mm, vinaceous to brown, minutely velutine, base rounded, margin hyaline, ciliate, apex hyaline, acute to obtuse, hairs hyaline. ***Flowers*** bisexual or staminate, zygomorphic, 0.7–0.9 cm diameter, pedicellate; pedicel 1.2–2.8 mm long, white, minutely velutine, hairs hyaline, patent and slightly elongate in fruit; floral buds 3.6–4.9 × 2.2–4.1 mm, broadly obovoid to subglobose, white, apex truncate to rounded, green; sepals 3.6–4 × 1.5–2.1 mm, narrowly ovate to elliptic, cymbiform, unequal, the uppermost external, broader and shorter than the others, fleshy, white, externally minutely velutine, hairs hyaline, internally glabrous, margin hyaline, glabrous to sparsely velutine, hairs hyaline, apex obtuse, green; petals 3.7–4.5 × 2.7–3.4 mm, trullate to obovate, the lowermost narrower than the others, white, base cuneate, margin entire, glabrous, apex obtuse to rounded; stamens 6, unequal, the anterior longer than the posterior, curved upwards, filaments 1.3–4.2 mm long, sigmoid, white, anthers 0.7–0.9 × 0.7–1 mm, anther sacs white, connectives quadrangular in the shorter stamens and rectangular in the longer, white; ovary 1.5–1.7 × 1.1–1.2 mm, ellipsoid, white, velutine, hairs hyaline, style 2.7–3.2 mm long, curved upward at the apex, white; stigma annular-capitate, papillate, white. ***Capsules*** 0.9–1.3 × 0.8–1.2 cm, subglobose to globose, green, sparsely reticulate, sparsely velutine, hairs hyaline. ***Seeds*** 3.6–3.9 × 2.9–3.2 mm, obconic to ellipsoid, medium to dark brown, testa scrobiculate; hilum longer than ½ the length of the seed; embryotega semidorsal; aril cream-colored, slightly translucent, thick.

##### Specimens seen (paratypes).

**BRAZIL. Espírito Santo**: Iúna, Serra do Valentim, trilha do Sr. Aristides, próximo à borda da mata, fl., 27 Jan 2012, J.P.F. Zorzanelli et al. 328 (VIES); floresta do Sr. Aristides, próximo à borda da vegetação, antes da primeira subida íngreme da trilha, fl., 15 Dec 2015, J.P.F. Zorzanelli 1391 (RB, VIES); floresta do Sr. Aristides, próximo ao início do zigue-zague da trilha, 20°21’ 56” S 41°28’ 26” W, fr., 31 Mar 2016, J.P.F. Zorzanelli 1505 (RB, VIES).

##### Etymology.

The epithet honors the collector of the type specimens, João Paulo Fernandes Zorzanelli, Brazilian botanist and dear friend of the authors. JPFZ is an active and prominent collector in the state of Espírito Santo, with collections currently focused on Serra do Valentim, the type locality of *S. zorzanellii*.

##### Distribution and habitat.

*Siderasis zorzanellii* is confined to the municipality of Iúna, Espírito Santo (Fig. 2). It occurs in the “Floresta Ombrófila Densa Montana” vegetation, at 1200–1350 m above the sea level, generally near disturbed sites, being less frequent in well-preserved areas. This could be related to its climbing habit and the need of more sunlight exposure then the rosette species of the genus. This pattern is common in other liana and vine groups, such as Bignoniaceae, Malpighiaceae, and Sapindaceae (Acevedo-Rodríguez, pers. comm.), especially evident in big families such as Asteraceae, where the primarily climbing genus *Mikania* Willd. is almost exclusively found at the edge of forests, along trails, and in disturbed areas (Oliveira 2015).

##### Phenology.

It was found in bloom from December to March and in fruit in March.

##### Conservation status.

*Siderasis zorzanellii* is very narrowly distributed, with an EOO of ca. 7.779 km^2^ and an AOO of ca. 300 km^2^. The subpopulations are small, with no more than 10 mature individuals each. Unlike for the rosette species in the genus, it is still uncertain if the two climbing species reproduce vegetatively through cloning. Flowering seems to be frequent, although fruits have been collected only once. Thus, following the recommendations from IUCN (2001), *S. zorzanellii* should be considered Critically Endangered [CR, A2abde+B1ab(iii, iv, v)+ B2ab(iii, iv, v)+C2a(ii)+D1+D2].

##### Affinities.

*Siderasis zorzanellii* is morphologically similar to *S. spectabilis.* Nevertheless, both species can be differentiated based on consistency of the leaf blades (chartaceous in *S. zorzanellii vs.* membranous in *S. spectabilis*), density of their pubescence (sparsely minutely velutine *vs.* minutely velutine), position of the inflorescences (terminal in the secondary branches or axillary in the older nodes of the primary branches *vs.* exclusively terminal in the primary branches), floral morphology (flowers 0.7–0.9 cm diameter, petals white, margins glabrous *vs.* flowers 1–1.3 cm diameter, petals dark mauve to vinaceous, rarely light pink or white, margins ciliate with non-moniliform hairs), and by their disjunct distribution (southern montane Espírito Santo state *vs.* northern montane Rio de Janeiro state).

## Final remarks

The present work adds four new species to *Siderasis*, along with the addition of new morphological characters that help clarify the circumscription of the group. *Siderasis* Raf. *emend.* M.Pell. & Faden may be uniquely characterized as comprising small perennial rosette herbs or robust perennial vines, with shoots determinate or indeterminate, leaves spirally- or distichously-alternate. The inflorescences are terminal or axillary, either a many-branched thyrse with alternate cincinni or reduced to a solitary cincinnus, cincinni always several-flowered. The flowers are chasmogamous, bisexual or male, actinomorphic or zygomorphic, and petals with glabrous margins or ciliated with non-moniliform hairs. The androecium is composed of 6 fertile stamens, filaments straight or sigmoid, anthers dorsifixed and extrorsely rimose, anther sacs semicircular, divergent, connectives expanded and quadrangular. In the gynoecium, the stigma is annular-truncate or annular-capitate, marginally papillate with unicellular papillae restricted to the margin of the stigmatic regions. Also, similar to *Dichorisandra*, the capsules are thick-walled, and the seeds are arillate, biseriate to partially uniseriate, with semidorsal or semilateral embryotega, and a C-shaped hilum. All species accepted by us are easily diagnosed by a unique and constant combination of morphological character states. Furthermore, each species can be easily separated based on their geographical distribution, since they are microendemics, with non-overlapping distribution areas (Fig. 2).

As indicated by several systematic studies in Commelinaceae (Evans et al. 2000, 2003; Hardy 2001; Wade et al. 2006; Zuiderveen et al. 2011; Hertweck and Pires 2014) and by the morphological evidence presented here and by Pellegrini (2017), the need to recircumscribe subtribe Dichorisandrinae is pressing. Aside from the cytological character of *x*=19 large chromosomes described by Jones and Jopling (1972) and hypothesized by Faden and Hunt (1991), no macro or micromorphological synapomorphies were found so far for subtribe Dichorisandrinae in its current circumscription. On the other hand, if subtribe Dichorisandrinae is recircumscribed to exclusively contain *Dichorisandra* and *Siderasis*, Dichorisandrinae *s.s.* can be easily morphologically characterized by its thick-walled capsules, the biseriate to partially uniseriate arillate seeds, semidorsal to semilateral embryotega, and C-shaped hilum. The lineage composed by *Geogenanthus* (*Cochliostema*+*Plowmanianthus*) needs to be formally recognized as a subtribe, and can be easily circumscribed by its petals with marginally fringed with moniliform hairs, and anthers sacs curved to spirally-coiled and appressed to each other. Phylogenetic studies using both nuclear and chloroplast sequences seem promising in elucidating phylogenetic incongruences in Commelinaceae (e.g. Burns et al. 2011). However, most phylogenetic in the family so far completely disregard morphological data, with the exception of Evans et al. (2000, 2003). Studies focusing on the systematics and recircumscription of Dichorisandrinae are currently being conducted, combining morphological and molecular data (Pellegrini et al., in prep.), and should shed some light on the evolution of the reproductive biology in the family.

## Supplementary Material

XML Treatment for Siderasis

XML Treatment for Siderasis albofasciata

XML Treatment for Siderasis almeidae

XML Treatment for Siderasis fuscata

XML Treatment for Siderasis medusoides

XML Treatment for Siderasis spectabilis

XML Treatment for Siderasis zorzanellii
